# Diverse Parental Experiences of Kangaroo Care in Neonatal Units Across Healthcare Systems: A Meta‐Synthesis

**DOI:** 10.1111/jan.17058

**Published:** 2025-05-19

**Authors:** Sirinthip Phuwayanon, Maria Healy, Breidge Boyle

**Affiliations:** ^1^ School of Nursing and Midwifery, Medical Biological Centre Queen's University Belfast Belfast UK; ^2^ Faculty of Nursing, Department of Paediatric Nursing Chiang Mai University Chiang Mai Thailand; ^3^ Mohammed Bin Rashid University Dubai UAE

**Keywords:** kangaroo care, kangaroo mother care, neonatal nurse, neonatal unit

## Abstract

**Background:**

Kangaroo Care is an effective practice recommended by WHO for newborns, especially preterm infants, to reduce mortality and morbidity and improve health outcomes. Understanding parents' experiences with Kangaroo Care is vital as it can significantly influence uptake and sustained practice; however, experiences may vary across healthcare systems.

**Aim:**

To explore parents' experiences of Kangaroo Care in neonatal units and to examine differences across international health systems.

**Design:**

A qualitative meta‐synthesis.

**Review Methods:**

A systematic search of the literature was carried out over seven databases, including CINAHL, MEDLINE ALL, EMBASE, PsycINFO, Maternity & Infant Care, Scopus and Cochrane Library. Qualitative studies published in English from 2010 to January 2024 were included. Data extraction and quality appraisal, using the CASP Qualitative Checklist, were undertaken. Meta‐synthesis of the included qualitative findings was carried out. The findings were reported following the Enhancing Transparency in Reporting the Synthesis of Qualitative Research (ENTREQ) guideline. The protocol was registered on PROSPERO (CRD42023483347).

**Results:**

Twenty‐five studies were included and four themes were identified: *parental fulfilment from Kangaroo Care, Hardship in Kangaroo Care practice, Roadblocks and difficulties in adopting and Building bridges to encourage and support Kangaroo Care.*

**Conclusion:**

This review underscores the multifaceted nature of parental experiences, including positive and challenging aspects, as well as significant barriers and facilitators that influenced Kangaroo Care implementation. By understanding these experiences and factors that hinder and enable, healthcare systems and professionals can better support and empower parents to improve the effectiveness of Kangaroo Care.

**Impact and Implications:**

Kangaroo Care is lifesaving, particularly in low‐income countries, but can be a challenge for parents providing it. By addressing deficiencies in infrastructure and resources, barriers can be minimised, thereby encouraging the practice of Kangaroo Care. This is especially important in lower‐middle‐ and low‐income countries where the practice is most effective and the practice is lowest.

**Patient or Public Contribution:**

This project is a meta‐synthesis; therefore, no patient or public contribution was deemed necessary.

## 1 Introduction

It is more than four decades since Kangaroo Care (KC) was proposed as a method of reducing neonatal mortality in poorly resourced areas (Rey and Martinez 1983). The provision of care for preterm and low‐birthweight infants still presents significant challenges for global healthcare systems. The World Health Organisation (WHO) now recommends KC as an evidence‐based, low‐technology and cost‐effective method for newborns to reduce mortality and severe neonatal morbidity, as well as improve the health outcomes for vulnerable newborns (WHO 2015; WHO 2021). The core components of KC include early, continuous, prolonged skin‐to‐skin contact, exclusive breastfeeding, early hospital discharge and adequate support with follow‐up at home (WHO 2003). There is strong evidence that KC positively impacts the physical health of preterm and low‐birthweight infants (Boundy et al. 2016; Sivanandan and Sankar 2023) and, of its holistic positive effect on both infants and parents, it offers multifaceted benefits, including well‐documented positive effects on parental mental health (Pathak et al. 2023; Saltzmann et al. 2021) meaning that it has been enthusiastically adopted even in highly resourced neonatal healthcare settings.

## 2 Background

Number three in the United Nations Sustainable Development Goals (SDGs) is universal health and well‐being, which is to a large extent dependent on the first zero poverty (Pradhan et al. 2017). However, although spending on health has generally increased globally, there are still wide divisions, with health care systems in low‐income countries (LICs) still often dependent on aid and with the health care provision of a country generally dependent on that country's overall income level (Chang et al. 2019).

Although KC has proven robust, effective and strongly recommended healthcare practice worldwide, its implementation for small and preterm neonates has varied considerably across different regions and healthcare systems. Global organisations have advocated for scaling up KC, which has achieved widespread acceptance. However, this intervention has not yet been fully integrated into all health systems (Cai et al. 2022). There is evidence that KC implementation varies significantly across countries with divergent income levels. Uptake is influenced by several factors, including healthcare infrastructure, government and policy support, cultural norm and contextual acceptance, resource availability and individual and family factors (Bayo et al. 2022; Seidman et al. 2015; Smith et al. 2017). Ironically, as the practice was first suggested for under‐resourced healthcare systems, its availability on a large scale in low‐ and middle‐income countries (LMICs) remains limited, with the practice being only moderately adopted in these regions (Dhage et al. 2023). However, while in the neonatal care setting in high‐income countries (HICs), KC tends to be an intermittent adjunct to other high‐technology treatments; in LICs, it is likely to be closer to the continuous, low‐technology treatment recommended by WHO (2003). Indeed, the terms Kangaroo Mother Care (KMC) and Kangaroo Care (KC) both involve skin‐to‐skin contact between a parent and a preterm or low‐birthweight infant; however, KMC is a more comprehensive intervention. It not only emphasises continuous and prolonged skin‐to‐skin contact but also promotes exclusive breastfeeding (WHO 2015; WHO 2022a; WHO 2022b). Conversely, KC is often utilised in a broader context to refer to intermittent skin‐to‐skin contact provided by either parent, typically the mother and father (Conde‐Agudelo and Díaz‐Rossello 2016). Skin‐to‐skin contact is regarded as a complementary practice to incubator care, but limited data are available on the scale‐up of KMC in HICs due to the widespread dependence on advanced technology in neonatal care (WHO 2023).

One of the critical gaps in the existing literature is limited qualitative research published on parents' experiences and the potentially divergent barriers and facilitators involved in providing KC with their infants in the neonatal unit across health systems. Understanding parents' experiences is crucial and may significantly impact the effectiveness and sustainability of its implementation. The variations across health systems may significantly influence how these differences affect parents' experiences with KC in the neonatal units. It is essential to consider conducting a further study with a more comprehensive review of qualitative research in this area. There is, therefore, a need to systematically synthesise the existing evidence and combine studies from HICs, MICs and LICs about parents' experiences, providing KC with a comprehensive understanding and identifying the factors that enable or hinder the adoption of this practice in the neonatal unit, accounting for variations in the health system.

Thus, this meta‐synthesis aims to address this gap by thoroughly analysing parents' experiences with KC across diverse health systems. The findings provide insights into parents' experiences with KC that may be valuable for how healthcare professionals and policymakers develop, improve and scale up KC across different contexts. Furthermore, consolidating international results from qualitative literature will contribute to enhancing high‐quality neonatal care, education and future research and positively impact this aspect of KC in neonatal units.

## 3 Aim and Research Questions

The aim of this review was to undertake a meta‐synthesis of current research on parents' experiences of KC with their infants in neonatal units across health systems.

## 4 Method

### 4.1 Design

Meta‐synthesis, a method used to identify, analyse, critically synthesise and integrate findings from multiple published qualitative studies, was employed using Sandelowski and Barroso's (2007) approach. This process involved several steps: identifying the research topic and formulating a focused review question, conducting a systematic literature search to retrieve relevant studies, performing a quality appraisal of the included studies, extracting the data and categorising the findings, meta‐summarising and synthesising the findings, followed by the presentation of the synthesis results. To ensure transparency in reporting, the synthesis of qualitative research followed the Enhancing Transparency in Reporting the Synthesis of Qualitative Research (ENTREQ) guidelines (Tong et al. 2012) (see Appendix S1). Furthermore, the protocol was registered in the International Prospective Register of Systematic Reviews (PROSPERO) with registration identification number CRD42023483347.

### 4.2 Search Strategy

A systematic search strategy was employed utilising the systematic review‐specific mnemonic PEO framework (Population, Exposure, Outcome), implemented to structure the review questions and construct the criteria for inclusion and exclusion to identify relevant papers (Muka et al. 2019). Seven electronic databases were searched, including CINAHL, MEDLINE ALL, EMBASE, APA PsycINFO, Maternity & Infant Care, Scopus and Cochrane Library. A comprehensive search strategy was formulated using MeSH headings and keywords aligned with the PEO framework, and truncations were tailored appropriately for each database. The keyword categories and search terms are detailed in Table 1. The integration of Boolean operators (OR/AND) to combine and link the search terms. Furthermore, the publication timeframe was limited between January 2010 and 2024.

**TABLE 1:** Systematic search terms strategy.

	P (population)	AND	E (Exposure)	AND	O (Outcomes)
Keywords	Women OR Women OR Parent* OR Mother* OR Maternal OR ‘New mother’ OR Father* OR Postnatal OR Postpartum OR ‘Birthing people’		‘Kangaroo Mother Care’ OR ‘Kangaroo Care’ OR ‘Kangaroo mother method’ OR ‘Skin‐to‐skin contact’ OR KMC OR KC OR SSC OR ‘Health system’		Experience* OR Attitude* OR Perception OR Emotions OR Feeling OR Facilitator* OR Barrier*

### 4.3 Eligibility Criteria

The eligibility criteria were established to determine the selection studies that should be included in the review, and which should be excluded, thereby minimising potential personal bias from researchers involved in the selection review process (Stern et al. 2014). Studies were selected based on the specific inclusion and exclusion criteria outlined in Table 2.

**TABLE 2:** Inclusion and exclusion criteria for included articles.

	Inclusion criteria	Exclusion criteria
Population and study focus	– All studies focusing on women or parents who have experienced Kangaroo Care in the neonatal unit	– Studies focused on maternity care providers or healthcare professionals' experiences with Kangaroo Care – Studies involved parents providing Kangaroo Care with their infants out of the neonatal unit (operating room and home)
Methodology	– Qualitative studies or mixed methods studies with reporting of qualitative data	– Quantitative methodologies
Research type Publication type	– Primary research studies – Full‐text articles published in a peer‐reviewed journal	– Secondary studies and non‐peer‐reviewed literature (e.g., commentary, dissertation, editorials, conference p and review articles)
Language	– Studies published in English	– Studies were not published in English

### 4.4 Study Selection

**FIGURE 1:**
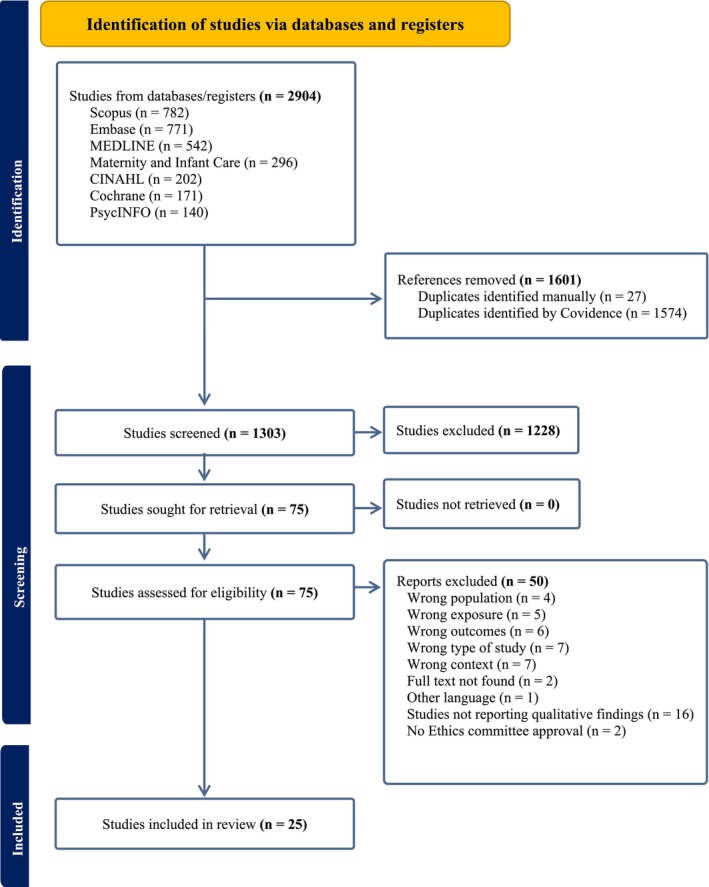
PRISMA flow diagram.

The findings from the search of the databases were directly imported into the Covidence systematic review software using the Endnote X9 reference management system, with all duplicate entries removed. Two reviewers selected studies independently by screening titles and abstracts for potential eligibility papers. After that, the full text was carefully considered in detail based on inclusion and exclusion criteria. Any disagreements or conflicts during this process were resolved through discussion with the third reviewer. The search results from specific databases, the study selection procedure and the reasons for exclusion were reported in the PRISMA flow chart, as outlined in Figure 1.

### 4.5 Quality Appraisal

The quality of evidence in the meta‐synthesis was assessed based on the research methodology approach. The qualitative studies were examined using the Critical Appraisal Skills Programme (CASP) checklist (Critical Appraisal Skills Program 2018). The 10‐item checklist of the CASP tool enables the assessment of whether the study meets the criteria by selecting ‘yes’, ‘no’ and ‘cannot tell’. Two authors independently evaluated, and the quality assessment results were cross‐checked by the reviewer team. Consensus was reached through discussion in cases of disagreement. All included studies ranged from moderate to high‐quality contributions. No studies were excluded from the analysis based on quality assessment, as all included studies were deemed to have the potential to offer meaningful insights. Details of the quality appraisal are presented in Table 3.

**TABLE 3:** Methodological quality assessment includes study.

Author and publication year	CASP 2018 for Qualitative research checklist										Quality appraisal Total score out of 10
	1. Clear statement of aims	2. Appropriate methodology	3. Appropriate research design	4. Appropriate recruitment strategy	5. Appropriate data collection method	6. Research relationship considered	7. Considered ethical issues	8. Rigorous data analysis	9. Clear findings	10. Value of research	
Blomqvist et al. (2011)	Yes	Yes	Yes	Yes	Yes	No	Yes	Yes	Yes	Yes	9
Noren et al. (2018)	Yes	Yes	Yes	Yes	Yes	Yes	Yes	Can't Tell	Yes	Yes	9.5
Olsson et al. (2017)	Yes	Yes	Yes	Yes	Yes	Yes	Yes	Yes	Yes	Yes	10
Lewis et al. (2019)	Yes	Yes	Yes	Yes	Yes	No	Yes	Yes	Yes	Yes	9
Saltzmann et al. (2021)	Yes	Yes	Yes	Yes	Yes	Can't Tell	Yes	Yes	Yes	Yes	9.5
Maastrup et al. (2018)	Yes	Yes	Yes	Yes	Yes	No	Yes	Yes	Yes	Yes	9
Dong et al. (2022)	Yes	Yes	Yes	Yes	Yes	Can't Tell	Yes	Yes	Yes	Yes	9.5
Arivabene and Tyrrell (2010)	Yes	Yes	Yes	Yes	Can't Tell	No	Yes	Can't Tell	Yes	Yes	8
Jesus et al. (2015)	Yes	Yes	Yes	Yes	Yes	No	Yes	Can't Tell	Yes	Yes	8.5
Lopes et al. (2020)	Yes	Yes	Yes	Yes	Yes	Can't Tell	Yes	Can't Tell	Yes	Yes	9
Pereira Viana et al. (2018)	Yes	Yes	Yes	Yes	Yes	Yes	Yes	Yes	Can't Tell	Can't Tell	9
Foong et al. (2023)	Yes	Yes	Yes	Yes	Yes	Can't Tell	Yes	Yes	Yes	Yes	9.5
Gunay and Coskun Simsek (2021)	Yes	Yes	Yes	Yes	Yes	No	Yes	Yes	Yes	Yes	9
Li et al. (2023)	Yes	Yes	Yes	Yes	Yes	No	Yes	Yes	Yes	Yes	9
Yue et al. (2020)	Yes	Yes	Yes	Yes	Yes	Can't Tell	Yes	Yes	Yes	Yes	9.5
Zeng et al. (2023)	Yes	Yes	Yes	Can't Tell	Can't Tell	No	Yes	Yes	Yes	Yes	8
Mpongwana‐Ncetani et al. (2023)	Yes	Yes	Yes	Yes	Yes	No	Yes	Yes	Yes	Yes	9
Ndou et al. (2021)	Yes	Yes	Yes	Yes	Can't Tell	No	Yes	Yes	Yes	Yes	8.5
Salimi et al. (2014)	Yes	Yes	Yes	Yes	Can't Tell	No	Can't Tell	Yes	Yes	Yes	8
Suza et al. (2020)	Yes	Yes	Yes	Yes	Yes	No	Can't Tell	Yes	Yes	Yes	8.5
Doukoure et al. (2022)	Yes	Yes	Yes	Yes	Yes	Can't Tell	Yes	Yes	Yes	Yes	9.5
Esewe and Phetlhu (2022)	Yes	Yes	Yes	Yes	Yes	Yes	Yes	Yes	Yes	Yes	10
Kourouma et al. (2021)	Yes	Yes	Yes	Yes	Yes	Yes	Yes	Yes	Yes	Yes	10
Asmare et al. (2021)	Yes	Yes	Yes	Can't Tell	Yes	No	Yes	Can't Tell	Yes	Yes	8
Naloli et al. (2021)	Yes	Yes	Yes	Yes	Yes	No	Yes	Yes	Yes	Yes	9

### 4.6 Data Extraction

Data extraction was undertaken independently by two reviewers. The data extraction describes the collection of necessary information from selected studies that could help address the review questions. The data extraction form captured the study characteristics, including the author's name, year of publication, study aims, design, methodology and method of data collection, participant details, primary findings and themes involving parental experiences of KC in the neonatal unit are summarised in Table 4.

**TABLE 4:** Characteristics of included studies.

No	Author, year country	Aims/objective of the study	Methodology/Data collection and data analysis	Setting	Participants and sample size (n)	Key findings (theme/subtheme)
1	Blomqvist et al. (2011) Sweden High‐income	To describe fathers' experiences of providing their preterm infants with KMC.	Qualitative descriptive research—Individual semi‐structured interviews **Data analysis** – Qualitative content analysis	NICU	Father of infants who were born at GA 28–33 weeks and 6 days. (*n* = 7) *Intermittent KC	One theme with four categories emerged. **Fathers' opportunities for being close to their infants facilitated the attainment of their paternal role in the NICU**. Handling unexpected situations: helping each other between father and mother and comparing them with other parents' situations. Feelings related to becoming a father: feelings related to KMC (positive experience, obvious role), feelings about and for the infant (special feeling, pride) and parental feelings (becoming a father, fatigue, tired). The father's experience of the division of roles between them and the NICU staff: positive about supportive, satisfied with the information, dissatisfied, conflict and variation about how they behaved and performed caregiving activities. Practical aspects: application of KMC (confidence) and accommodation (uncomfortable, assistance from relatives and friends).
2	Noren et al. 2018 Sweden High‐income	To describe Swedish mothers' experiences of KMC.	Qualitative descriptive study—Individual interviews **Data analysis** – Qualitative content analysis	NICU	Mothers of infants who were born at PMA 28–33 weeks and 6 days. (*n* = 13) *Intermittent KC	Three content areas and categories were identified based on the definition of KMC. **Skin‐to‐skin contact:** separation or closeness (supportive: family room, socialise with other parents and barriers: lack of privacy, crowed people), becoming a mother (closeness, parental role), taking turns for continuously SSC, practical aspects (exhausting, lack of comfort) and experiences of KMC (positive attitudes) **Breast milk as nutrition, if possible:** expression of breast milk and breastfeeding **Early discharge from the NICU:** early discharge and closeness at home
3	Olsson et al. (2017) Sweden High‐income	To describe fathers' experiences of skin‐to‐skin contact with their premature infant.	Qualitative study—Semi‐structured interview **Data analysis** – Qualitative content analysis (Deductive)	Neonatal unit	Fathers of premature infants should have provided SSC (*n* = 20) *Intermittent KC	The categories were represented by Anderzen‐Carlsson's model **A restorative experience** Feeling good: a heart‐warming experience, relieving emotional suffering, a rewarding experience, a natural instinct, a learning experience, finding a role, improved self‐esteem, a feeling of control and a supportive environment (staff, privacy space, family, social support) Doing good: a way of knowing and understanding and important for infant Becoming us: a bonding experience and intimate togetherness (taking turns) **An energy‐draining experience** Feeling exposed: environment as an obstacle, the physical and emotional burden (exhaustion, lack of sleep, afraid, anxiety), incongruence between wishes and demands, uncertainty about the purpose of and own skill in providing SSC. Hurting others: fear of hurting and feeling insufficient toward the family
4	Lewis et al. (2019), USA High‐income	To understand barriers and facilitators to provision of KMC in the NICU in a high‐income country context.	Qualitative descriptive study—Semi structure interviews **Data analysis** – Thematic analysis approach (Inductive)	NICU	Mothers of preterm infants. (*n* = 20) *Intermittent KC	The themes are organised by each type of factor within Andersen's model. **Predisposing factors** Stress of preterm birth: a feeling of being overwhelmed, unexpected hospitalisation, emotionally and physically taxing, feeling shocked, guilty and stressed Difficulty recovering from birth: physical recovery limited self‐care and influenced mother's ability to Baby's' care and spend time in the NICU **Perceived need factor** Perceptions of KMC were primarily shaped by the knowledge and guidance provided by nurses. The benefits of KMC, such as bonding and a strong sense of joy and love, were key factors in mothers' experiences. Fear for child's health: fear of KMC making the child cold and stressed, harm due to small size, disturbing medical equipment and concern monitoring of child's progress **Enabling factors** Maternity leave or the lack thereof, was a significant enabling factor. Many mothers faced financial struggles when they had to take unpaid leave or had limited or no maternity leave options. Accessing the hospital: concern about accessing adequate and affordable accommodation near the NICU, managing transport to the hospital and support from the hospital social worker (emotional resources)
5	Saltzmann et al. (2021) California, USA High‐income	To explore parental understanding, parental perception of experiences and parental views on the key factors that help and hinder their ability to take part in KC.	Mixed‐method study—Observational—The survey consisted of 12 questions with a mix of Likert scale and open‐ended free‐text responses **Data analysis** —Descriptive statistics —Thematic analysis (Inductive)	NICU	Parent with at least 1 previous KC experience (*n* = 50) *Intermittent KC	Six main themes emerged. Wanting more information Barriers: fear, anxiety and afraid of hurting, baby's medical issues, pain, fatigue, NICU environment and negative impressions of staff Parental mental health: KC help their emotional well‐being (positive experience, improved bonding, feeling like a mother) and physical well‐being Nursing support: positive, emphasised and want more nursing support, consistency around holding babies Needed improvements: environment, flexibility of touch, better communication, emotional and physical support. The effects of KC on parents are positive experiences, helping emotional well‐being, improving bonding and providing a sense of parenthood.
6	Maastrup et al. (2018) Denmark High‐income	To explore parents' immediate experiences of skin‐to‐skin contact with their extremely preterm infant	Qualitative descriptive methodology—Semi‐structured interview, face‐to‐face interview **Data analysis** – Thematic analysis (Inductive)	NICU	Parents of extremely preterm infant (*n* = 13) *Intermittent KC	Three themes constituted consecutive stages in the process of skin‐to‐skin contact. Overcoming ambivalence through professional support and personal experience: conflicting feelings and needs (concern about harming and safety, feeling close) and gaining confidence through encouragement (essential role of nurses to promote SSC) Proximity creates parental feelings and an inner need to provide care: immediate happiness (ambivalent: happy and sad) and formation of parental identity (being a parent) Feeling useful as a parent and realising the importance of skin‐to‐skin contact: taking on parental responsibility and important difference for the infant Bonding is beneficial regardless of survival: the value of bonding and close physical contact.
7	Dong et al. (2022), Australia High‐income	To explore fathers' views and experiences of providing KC to their baby cared for in an Australian NICU.	A qualitative descriptive approach—Semi‐structured interview **Data analysis** – Thematic analysis	NICU	Fathers who had experienced at least one episode of KC (*n* = 10) *Intermittent KC	Three key themes and eight subthemes emerged from the views and experiences **Positive psychological connection** Feeling nervous: fragile babies, unfamiliar with equipment, fear of possible emergencies and excitement Experiencing calm and relaxation: ease, satisfaction and relaxation Enjoying connection: feeling of closeness to baby, affection and happiness in the father Engaging with confidence: positive emotion, confidence to engage and provide KC, having opportunity to learn and connection with baby **Embracing KC** Experiential learning: eager to seek further KC knowledge and wishing in relation to promoting father‐infant KC Reimagining the paternal caregiving: physical closeness with babies and understanding of the parental role **Challenges to father‐infant KC** Competing priorities: manage conflicting needs to provide KC Physical discomfort: characteristic of masculinity and uncomfortable
8	Arivabene and Tyrrell (2010) Brazil Upper‐middle income	To describe the mothers' experiences in Kangaroo mother method. To analyse the mothers' experiences in the light of Kangaroo mother method principles.	Qualitative research – Interviews – Focus group discussion	NICU	Mothers of low‐birthweight infants. (*n* = 13) *Not specified type of KC	Themes categories were constructed. Benefits of experiences in the kangaroo mother method: the infant's survival and recovery: fear, anxiety and feeling of guilt but benefits for infant's survival The mothers' daily life modified by the kangaroo mother method: afflicted by abandoning their other children and husband, complicating relations, task overload Valuation of affection among relatives when the family members effectively participated in KMM
9	Jesus et al. (2015) Brazil Upper‐middle income	Identifying the father's perception about the experience of the Kangaroo Method.	Qualitative approach—Semi‐structured interview **Data analysis** – Content analysis	Maternity	Fathers of preterm and/or low birthweight and experiencing the Kangaroo (*n* = 6) *Intermittent KC	Thematic categories emerged Feelings and sensations of the father who experiences the Kangaroo Method: positive experiences (family strengthening, warm feeling and satisfaction) and negative feelings (psycho‐emotional suffering, fear and insecure) Approach to the (un) known: knowledge and ignorance is the lack of information and clarification of the Kangaroo method The construction of the paternal bond: positive influence, favouring of a loving relationship and feeling about being a father
10	Lopes et al. (2020), Brazil Upper‐middle income	To describe the fathers' experiences using the kangaroo position with their low‐birthweight newborns	Qualitative approach—Semi‐structured interviews **Data analysis** – Thematic content analysis	NICU	Fathers that used KP participated (*n* = 5) *Intermittent KC	Three categories merged to identify the fathers' experiences with the Kangaroo position. The ambivalence of feeling: positive feeling, love and affection, sense of protection and feeling of fear of hurting and insecurity The ease and difficulty experienced: The fathers had a comfortable and wonderful experience with the nursing team despite the physical accommodations presenting some challenges. The strengthening of the father‐child bond
11	Pereira Viana et al. (2018), Brazil Upper‐middle income	Describe and analyse the experience of mothers of premature infants in the Kangaroo Mother Method	Qualitative approach—Semi‐structured individual interview **Data analysis** – Content analysis	A public maternity hospital	Mothers who had preterm children in the Kangaroo Mother Method (*n* = 15) *Not specified type of KC	The categories emerged **Experiencing and Learning the Kangaroo Mother Method** Knowledge about the method and benefit**s**: the needs of more effective orientation Mother's experiences and perceptions: positive experiences, understood the importance of the method and positively interfere with infant's relationship Emotional struggles and physical fatigue Limited information of method: mothers were able respond about KMM in a simple way without much depth
12	Foong et al. (2023) Malaysia Upper‐middle income	To gain deeper insight into factors influencing the uptake of KMC practice	Qualitative study—Semi‐structured interviews **Data analysis** – Thematic analysis	The neonatal unit and obstetric unit	Parents who had prior KMC introduction or KMC experience (*n* = 9) *Intermittent KC	The finding for each variable uses Triandis' theory of interpersonal behaviour as a framework. The habit component: earlier introduction to the KMC concept would have better prepared parents to do KMC, and traditional confinement is difficult to do KMC. The intention component: emotional ranging from positive and negative feelings (warmth, happiness, contentment, scared to being uncertain, stressful for monitoring the baby's well‐being and feeling embarrassed of doing KMC in open ward) Social: society expects parents to do the best for babies, beneficial and opportunity to learn and practice baby care Cognition: KMC returned a sense of parenthood, the opportunity to be with their baby and exhaustion to do KMC Facilitating factors: special privileges, lack of facility to practice, lack of support from HCP and education materials were inadequate
13	Gunay and Coskun Simsek (2021) Turkey Upper‐middle income	To investigate the emotions and experiences among fathers who apply kangaroo care in NICU.	Qualitative design – Face‐to‐face, in‐depth interviews **Data analysis** – Content analysis (Inductive)	NICU	Fathers of preterm infants (*n* = 12) *Intermittent KC	Three main themes and their six subthemes emerged **Emotions of being a father** Feeling the warmth and scent of the baby: feeling that they were their baby's father and connection Emotional that the baby belongs to one: feel fatherhood **Confidence in fathering roles** Self‐confidence: increase the father's belief in performing fatherhood role Caring for the baby: responsibility for their babies **Happiness in the new father role** Seeing the baby calm down Hugging the baby and touching the baby's skin: surprised and beautiful feeling
14	Li et al. (2023) China Upper‐middle income	To examine the experience of early skin‐to‐skin contact and non‐nutritive comfort sucking of mothers of hospitalised preterm infants	Qualitative study—Semi‐structured in‐depth interview **Data analysis** – Inductive topic analysis	NICU	Mothers of preterm infants (*n* = 18) *Intermittent KC	Five themes about skin‐to‐skin contact were identified Theme 1: alleviation of maternal anxiety and fear during mother‐infant separation (feel relieved, happy) Theme 2: Reshaping the maternal role (becoming closer, becoming a spiritual mother, being a mother) Theme 3: Promotion of active breast pumping Theme 4: Enhances the mother's willingness to actively breastfeed Theme 5: Building the maternal confidence in baby care
15	Yue et al. (2020) China Upper‐middle income	To establish a conceptual framework to analyse the barriers and facilitators to KMC scale‐up and provide recommendations for KMC adoption in Chinese hospitals	Qualitative Study—clinical observation and semi structure interviews **Data analysis** – A framework analysis	NICUs and postnatal wards	10 parents who had experience with KMC 18 nurses 10 doctors (*n* = 38) *Intermittent KC	A conceptual framework categorised the different levels of barriers and facilitators for KMC adoption. Cultural level: Barriers (postpartum confinement and grandparents' resistance to KMC) and Facilitators (strong family support) Hospital level: Barriers (lack of private space, fear of nosocomial infection and parents monitoring work) and a powerful Facilitator (the influential leadership of the hospital's administration and the creation of a supportive community) Parental level: Barriers (lack of private space, maternal guilt associated with preterm birth and facing medical monitors) and facilitators (benefits to mothers and newborns) Financial level: Facilitators (parents with good financial status)
16	Zeng et al. (2023) China Upper‐middle income	To understand fathers' experiences and inner feelings about participating in kangaroo care of premature infants	Qualitative study—Semi‐structured interview **Data analysis** – Colaizzi phenomenological method	NICU	Fathers of premature infants who carried out KFC (*n* = 12) *Intermittent KC	The findings identified two main themes **Positive experience** Creating the feeling of being a father: a sense of reality and a real sense of being a father Deepening the correlation between the father and the child: infants gradually change from strangers to trusting their fathers and deepen the bond. Enhancing confidence in parenting: more skilled, confidence, increased ability to take care of their babies Reducing stress and gaining happiness Internalising care: better understand the hardship of mother **Negative experience:** the father had never heard of and did not know how to do KFC at the beginning and was ambivalent (afraid of hurting, worried and not skilled)
17	Mpongwana‐Ncetani et al. (2023) South Africa Upper‐middle income	To explore the experiences of Xhosa mothers providing KMC to their preterm babies	Qualitative study—Semi‐structure interviews **Data analysis** – Thematic analysis	KMC ward	Mothers attending the KMC ward (*n* = 10) *Including intermittent and continuous KC	The findings are organised into four main themes. KMC is beneficial to their infants but a foreign concept Distress in the KMC ward: physical demands related to KMC (feeling close, impairing sleep, overwhelmed), the mental health of KMC mothers (worry and anxiety about babies' health) and stressful environment The missing umbilical cord: cultural experiences of mothers in the KMC ward and ambivalent about cultural practices The KMC village: interpersonal relations in the ward with nurses, doctors and between mothers (source and emotional support)
18	Ndou et al. (2021) South Africa Upper‐middle income	To document lived‐in experiences of mothers providing KMC in Vhembe District hospitals	Phenomenological approach—In‐depth face‐to‐face interview	KMC unit	Mothers who gave birth to preterm babies and were providing KMC (*n* = 13) *Continuous KC	Three main themes and subthemes emerged **Knowledge of mothers about KMC** The practice of KMC: position of babies The rationale behind practising KMC: providing warmth and opportunity to be close to infant Benefits of practising KMC **Challenges of providing KMC** Strained relationships between family members Physical challenges: fatigue Inadequate hospital amenities: basic amenities and encourage dirtiness **Support of the mothers** Emotional support: nurses and family members
19	Salimi et al. (2014) Iran Upper‐middle income	To determine experiences of mothers having premature neonates about kangaroo care	Qualitative research—Focus group discussion **Data analysis** – Content analysis: conventional interpretation approach	NICU	Mothers having premature neonates (*n* = 12) *Not specified type of KC	The findings emerged in two significant categories. Mothers' experiences about advantages of kangaroo care in interaction with neonates: a sense of satisfaction and tranquillity, motherhood, alleviation of fear and a deep feeling of attachment The feeling of physical and mental healthiness of neonate: the feeling changes (decreased crying, adjustment of vital signs and babies being more tranquil) and obstacle of KC (insufficient instruction, inappropriate atmosphere, absence of fathers and presence of medical equipment)
20	Suza et al. (2020) Indonesia Upper‐middle income	To describe the barriers and mothers' experience in implementing KMC during hospitalisation with LBW neonates.	Qualitative research with descriptive phenomenology approach – Semi‐structured questionnaire interviews **Data analysis** – Colaizzi method	Perinatology unit	Mothers who have babies with LBW and having experience in KMC (*n* = 30) *Not specified type of KC	Five themes emerged based on the experiences of mothers in implementing KMC. Feeling scared that something would happen: KMC was strange and frightening, scary and afraid of hurting the baby. Improving the survival and recovery of the baby: pay attention to and fulfil the baby's needs Increase bonding (emotional bond) between mother and baby: hugging, talking, playing and breastfeeding. Mood disturbances: higher levels of stress and anxiety Environment as an obstacle: unavailability of admission room, lack of privacy, feeling limited and discomfort, and uncertainty about the ability to do KMC
21	Doukouré et al. (2022) Cote d'Ivoire Lower‐middle income	To assess mothers' acceptance of KMC based on their perceptions of care	Qualitative Study – Semi ‐structure interviews **Data analysis** – A framework analysis (Deductive: TFA driven)	KMC unit (A part of NICU)	Mothers of preterm and low birthweight who received KMC (*n* = 32) *Continuous KC	The findings are presented for the Theoretical Framework Acceptability (TFA) **Affective attitude:** very feeling positive, strengthens the bond **Perceived effectiveness:** understood as the extent to which a practice is perceived as likely to achieve its purpose, and perceived KMC is an effective method for child survival. **Burden:** focus on the perceived effort to participate in KMC and simple method **Self‐efficacy:** confidence to participate in KMC, lack of family support and fatigue due to other tasks impede the continuation of KMC **Intervention coherence:** understood as the extent to which participants recognise the aim of KMC. **Opportunity costs:** understood as the extent to which benefits, advantages and value (maternity leave, housewife, self‐employed) **Ethicality:** KMC were perceived to be a good fit with the mothers' value system, but some cultural beliefs are difficult to accept KMC
22	Esewe and Phetlhu (2022) Nigeria Lower‐middle income	To investigate the challenges faced by parents of preterm and low‐birthweight infants in the uptake of KMC.	Exploratory qualitative research—Semi structure interviews **Data analysis** – Thematic analysis	The neonatal special care baby unit	Mothers of preterm/LBW infants who had practiced KMC (*n* = 13) *Intermittent KC	Main themes with subthemes and three categories were generated. **Challenges experienced with practice** Deterrents to acceptance of KMC practice: gossip and ridicule from friends, discouragement from others, nurses' attitude and lack of privacy Shortage of resources: human, physical and financial Impact of challenges: a sense of despair, inadequate information and limited opportunity to practice KMC.
23	Kourouma et al. (2021) Cote d'Ivoire Lower‐middle income	To investigate barriers and facilitators of KMC and proposed solutions to improve KMC implementation	Qualitative Study—Semi‐structured Interview **Data analysis**—Thematic analysis (Inductive and Deductive)	KMC unit (A part of NICU)	32 Mothers who were receiving or received KMC 12 healthcare providers involved in KMC implementation (*n* = 44) *Continuous KC	The Consolidated Framework for Implementation Research (CFIR) construction identified the domains as barriers and facilitators. Intervention characteristics: Facilitator of KMC is a low‐cost practice Outer setting: Barriers are lack of community awareness, beliefs that led to lack of information on KMC and the father's resistance (difficulty to accept being separated from spouse and child) Inner setting: Barriers are increased workload and lack of food, space and supplies for admitted mother. Facilitators are strong leadership and training of HCP Characteristics of individuals: Facilitators are mothers' adherence to KMC, good relationship, feeling supported and listening skills of HCP that the way they behaved, became familiar and facilitated their uptake of KMC
24	Asmare et al. (2021) Ethiopia Low‐income	To explore the perceived enablers and barriers of KMC among mothers and nurses	Phenomenological study—Semi‐structured open‐ended questionnaires **Data analysis** – Thematic analysis	NICU	13 mothers of neonates receiving KMC and 7 nurses working at the KMC unit. (*n* = 20) *Not specified type of KC	Three primary themes and subcategories concerning perceived facilitators and barriers to the practice of KMC among mothers and nurses. **Support of mother:** family support, healthcare staff and setting (private and quiet room, workload, limited resources and facilities) and no culture and religion barrier in the Ethiopian community **Medical condition:** maternal and neonatal medical condition (fear for neonate's health, pain of wound from caesarean section) **Support to nurses:** caregiver and healthcare staff and setting
25	Naloli et al. (2021) Uganda Low‐income	To identify the supporting factors and hindrances to the effective performance of KMC practice among mothers/caretakers in the NICU	Qualitative methods—In‐depth interviews	NICU	17 Mothers and 3 caretakers with preterm neonates who had been initiated into KMC (*n* = 20) *Continuous KC	The themes and subthemes were categorised according to the experiences **Theme 1:** Facilitators to KMC practice Subthemes: general knowledge about KMC and its benefits, ability to have a substitute KMC provider (family members), mothers agreeing that KMC promotes breastfeeding and infant bonding, improved monitoring of unstable neonates and well‐being (sense of responsibility), positive attitude (happy), family support, Male involvement (partner and relative), Medical/Health workers' support and peer counselling. **Theme 2:** Barriers to KMC practice. Subthemes: inadequate health workers support, the attitude of health workers, lack of male involvement, environmental hindrances (infrastructure challenges), maternal health status (physical and mental health), stress, multiple roles, religion, culture and tribe (prohibition), infant related hindrances (misconception about preterm) and lack of enough time to anticipate and psychologically prepare for such a time

### 4.7 Data Synthesis

The meta‐synthesis was derived by rigorously synthesising the findings from the different original qualitative studies through an iterative process to produce generalisable results and develop new knowledge (Finfgeld‐Connett 2010; Walsh and Downe 2005). A thematic analysis approach, as described by Braun and Clarke (2019), was employed to conduct the reflexive narrative synthesis of the qualitative data and consolidate the findings through meta‐aggregation, focusing on emphasising participant experiences. The thematic matrix entailed extracting findings from each primary study, initially coding them into categories, and subsequently synthesising them into meta‐themes reflecting distinct parents' experiences of KC. The reflexive thematic analysis was performed to systematically code the data obtained from the included studies to comprehensively convey meaning, focusing on examining themes within data. The authors read the articles multiple times to familiarise themselves with the content of each paper and conducted line‐by‐line initial coding of the findings of all primary studies. All relevant text from the findings was extracted and entered into the QSR NVivo software. The code development process involved deep and prolonged data immersion, discussion and comparison of interpretations to capture the meaning of the data and context. Codes were organised into related areas to construct themes through an iterative process. The similarities and differences between the codes were initially organised in a hierarchical structure. The potential emerging themes were compared within and across studies to answer the review questions. Finally, the final themes were derived inductively and were then summarised to go beyond the findings of the original study. The themes and subthemes were then reviewed through discussion and revision among the reviewer team.

## 5 Findings

### 5.1 Search Outcome

The search resulted in 2904 articles that were obtained through seven database searches. After removing duplicates, 1303 studies underwent screening based on titles and abstracts. Subsequently, 75 articles were retrieved for full‐text assessment for eligibility to be included in the review. Following careful examination of each paper with the full text retrieved, 25 articles met the proposed criteria for inclusion. The result is illustrated in the PRISMA flow diagram (Figure 1).

### 5.2 Characteristics of Included Studies

A total of 25 studies were included that met the eligibility criteria. The studies had a wide diversity of study designs, predominantly 24 utilising qualitative methodologies and one using mixed‐method studies. The studies encompassed a range of studies from diverse global regions, indicating a global interest in KC in neonatal care practices. The countries where studies were conducted include Brazil (*n* = 4), Sweden (*n* = 3), China (*n* = 3), the USA (*n* = 2), Cote d'Ivoire (*n* = 2), South Africa (*n* = 2), Ethiopia (*n* = 1), Australia (*n* = 1), Nigeria (*n* = 1), Malaysia (*n* = 1), Turkey (*n* = 1), Uganda (*n* = 1), Denmark (*n* = 1), Iran (*n* = 1) and Indonesia (*n* = 1). The studies were categorised according to countries' income levels: 13 in upper‐middle‐income countries (UMICs), 7 in high‐income countries (HICs), 3 in low‐middle‐income countries (LMICs) and 2 in low‐income countries (LICs). Most studies employed qualitative descriptive approaches derived from semi‐structured interviews and focus group discussions. The most common analysis methods to gather in‐depth insights were thematic analysis and content analysis. Details of characteristics are provided in Table 4.

### 5.3 Themes

This review presents four themes and eleven subthemes derived inductively from the data: (1) Parental Fulfilment from Kangaroo Care, (2) Hardship in Kangaroo Care practice, (3) Roadblocks and difficulties in adopting Kangaroo Care and (4) Building bridges to encourage and support Kangaroo Care. Details on how the themes relate to each paper and identified subthemes are presented in Table 5.

**TABLE 5:** Matrix of included studies and themes identified in each study.

Themes	Paper references																								
	HICs							UMICs													LMICs			LICs	
	1	2	3	4	5	6	7	8	9	10	11	12	13	14	15	16	17	18	19	20	21	22	23	24	25
Theme 1: Parental Fulfilment from Kangaroo Care																									
Emotional well‐being	✓	✓	✓	✓	✓	✓	✓	✓	✓	✓		✓	✓	✓		✓									✓
Strengthening bond			✓	✓	✓	✓			✓	✓	✓					✓			✓	✓	✓				✓
Empowering parenthood	✓	✓	✓		✓	✓	✓		✓			✓	✓	✓		✓	✓		✓		✓				✓
Theme 2: Hardship in Kangaroo Care practice																									
Emotional suffering			✓	✓		✓	✓	✓	✓	✓	✓	✓				✓	✓			✓			✓	✓	✓
Physical burden	✓	✓	✓				✓				✓	✓					✓	✓							✓
Theme 3: Roadblocks and Barriers in adopting Kangaroo Care																									
Institutional and professional barriers	✓	✓	✓		✓				✓	✓		✓			✓		✓	✓	✓	✓		✓	✓	✓	✓
Cultural and social barriers												✓			✓		✓		✓		✓	✓	✓		✓
Physical barriers and medical issues	✓		✓	✓	✓		✓								✓		✓		✓		✓			✓	✓
Theme 4: Building bridges to encourage and support Kangaroo Care																									
Healthcare systems support	✓	✓	✓	✓	✓	✓				✓	✓	✓			✓		✓	✓					✓	✓	✓
Social and psychological Support		✓	✓	✓											✓		✓								✓
Strengthen family support	✓	✓	✓					✓							✓			✓						✓	✓

Abbreviations: HICs, high‐income countries; LICs, low‐income countries (based on World Bank countries classifications); LMICs, lower‐middle‐income countries; UMICs, upper‐middle‐income countries.

#### 5.3.1 Theme One: Parental Fulfilment From Kangaroo Care

The theme explores the emotional aspect of both continuous and intermittent KC that reflects the positive experiences and perceptions of parents providing KC for their infants in the neonatal unit. Parents overwhelmingly describe a multitude of positive emotions across various domains, reflecting a profound sense of fulfilment for parents as they provide nurturing care for their vulnerable infants. The analysis reveals three subthemes: emotional well‐being, strengthening bonds and empowering parents.

##### 5.3.1.1 Subtheme: Emotional Well‐Being

This theme highlights the positive experiences and perceptions of parents when engaging in KC. Parents described experiencing a profound sense of overwhelming love, affection and happiness (Arivabene and Tyrrell 2010; Dong et al. 2022; Foong et al. 2023; Gunay and Coskun Simsek 2021; Jesus et al. 2015; Lewis et al. 2019; Lopes et al. 2020; Maastrup et al. 2018; Naloli et al. 2021; Olsson et al. 2017; Saltzmann et al. 2021; Zeng et al. 2023). Parents experienced heightened feelings of closeness with their infant (Blomqvist et al. 2011; Dong et al. 2022; Gunay and Coskun Simsek 2021; Li et al. 2023; Lopes et al. 2020; Maastrup et al. 2018; Noren et al. 2018; Olsson et al. 2017). Furthermore, many parents conveyed experiences of warmth and tranquillity (Dong et al. 2022; Foong et al. 2023; Gunay and Coskun Simsek 2021; Jesus et al. 2015; Maastrup et al. 2018; Noren et al. 2018; Olsson et al. 2017). These meaningful responses highlight the deeply affective nature of KC as one parent described their experience:

You are happier afterwards, because it is such a difficult situation to have a baby, where the future uncertain. It's (the skin‐to‐skin contact) a very positive experience. In fact, there is nothing that has changed in her health, I'm just happier(Maastrup et al. 2018, 549).

Seven studies captured fathers' experiences while participating in intermittent KC conducted in UMICs and HICs, including Sweden, Brazil, Turkey, Australia and China. Fathers described feeling a unique sense of happiness, affection, closeness, warmth and emotional fulfilment as they nurtured their infants through KC (Blomqvist et al. 2011; Dong et al. 2022; Gunay and Coskun Simsek 2021; Jesus et al. 2015; Lopes et al. 2020; Olsson et al. 2017; Zeng et al. 2023). One father stated:

A love, I guess, that I hadn't experienced until then. An affection, an inner peace, I did it! To be there with her. To want to hold her, to look at her, to cuddle her. I think I mostly felt a kind of peace, you know? Peace and affection(Lopes et al. 2020, 4).

Furthermore, the study by Olsson et al. (2017) conducted in Sweden highlighted that KC fostered a wonderful feeling, a sense of emotional satisfaction, value in monitoring infant development and delight in watching their infants become stable and calm.

##### 5.3.1.2 Subtheme: Strengthening Bond

When first practising KC with infants, some parents may initially experience feelings of anxiety and unfamiliarity. However, consistent implementation has been linked to strengthened bonds and attachment between parents who feel a deeper connection with their infants through KC (Jesus et al. 2015; Lewis et al. 2019; Lopes et al. 2020; Naloli et al. 2021; Pereira Viana et al. 2018; Salimi et al. 2014; Saltzmann et al. 2021; Suza et al. 2020; Yue et al. 2020; Zeng et al. 2023). Several studies (Doukouré et al. 2022; Maastrup et al. 2018; Olsson et al. 2017) have highlighted that parents described feeling a sense of closeness when engaging in direct physical contact with their infants, which gradually reinforces the emotional connection and fosters a stronger parent‐infant relationship. According to research in Denmark by Maastrup et al. (2018), KC is a fundamental nurturing practice that emerged and strengthened the bonding experience. Similarly, a Uganda study (Naloli et al. 2021) underscored that mothers recognised KC promotes infant bonding, as one mother described:

When you place the baby on the chest, it increases the bonding and love between the mother and the baby(Naloli et al. 2021, 7).

Fathers gradually experienced a strengthening of their bond with infants as they became more familiar with each other through KC practice (Jesus et al. 2015; Lopes et al. 2020; Olsson et al. 2017; Zeng et al. 2023). Holding an infant close to their chest and feeling their heart beating contributed to an instinctual sense of attachment and responsibility in the fathers (Olsson et al. 2017). Consistent with the findings of Zeng et al. (2023) in Brazil, KC established a stronger emotional bond and connection between father and child. This study emphasised that these bonding experiences emotionally benefit the father's well‐being and positively impact the infants. As one father described:

We are with him to look after it, and he recovers as quickly as possible. I feel a very strong bond that I am having with him. This is very good for him and for us.(Jesus et al. 2015, 8546).

##### 5.3.1.3 Subtheme: Empowering Parenthood

The implementation of KC has demonstrated a noteworthy impact on the perceived sense of motherhood or fatherhood (Blomqvist et al. 2011; Foong et al. 2023; Gunay and Coskun Simsek 2021; Jesus et al. 2015; Maastrup et al. 2018; Noren et al. 2018; Salimi et al. 2014; Saltzmann et al. 2021; Zeng et al. 2023), fostering a robust identity as a caregiver and a deepened sense of purpose in their parental role (Dong et al. 2022; Doukouré et al. 2022; Maastrup et al. 2018; Mpongwana‐Ncetani et al. 2023; Naloli et al. 2021; Olsson et al. 2017), thereby contributing to the improved infants' well‐being. Additionally, engaging in KC has been found to contribute to parents developing greater confidence in their abilities to care for their infants (Dong et al. 2022; Gunay and Coskun Simsek 2021; Li et al. 2023; Olsson et al. 2017; Zeng et al. 2023). The quote from a parent exemplified this:

While doing KMC, I finally felt like a mother. I have longed to hold him(Foong et al. 2023, 6).

Five studies found that KC plays a significant role in fostering fathers feeling of truly becoming real fathers and strengthening their sense of fatherhood (Blomqvist et al. 2011; Dong et al. 2022; Gunay and Coskun Simsek 2021; Jesus et al. 2015; Zeng et al. 2023). Specifically, fathers expressed special feelings and a sense of pride while engaging in KC (Blomqvist et al. 2011; Olsson et al. 2017). One father noted the importance of practice, stating,

I think that was the most thing that I find with the cuddling, helping me to get closer to her and doing my part as a father, helping out more(Dong et al. 2022, 8).

Furthermore, numerous studies have underscored that fathers often feel a stronger sense of identity, purpose and responsibility in parental roles when holding their baby in their arms. These studies have revealed that participation in KC empowered fathers by reinforcing their role and increasing confidence in their abilities as primary caregivers (Dong et al. 2022; Gunay and Coskun Simsek 2021; Olsson et al. 2017; Zeng et al. 2023). A father from a study conducted in Turkey stated,

I felt responsible; there is now a baby in my life I am responsible for. I became aware that we are family. My self‐confidence increased(Gunay and Coskun Simsek 2021, 843).

#### 5.3.2 Theme Two: Hardship in Kangaroo Care Practice

KC practice provides significant emotional benefits for parents, but it also presents various hardships that can complicate parental experiences more difficult. The research points out that parents' experiences have a range of mixed emotions towards KC, experiencing both happiness and struggling with anxiety and psychological stress. This theme delves into the emotional struggles that parents encountered, reflecting subthemes, including emotional and physical burdens.

##### 5.3.2.1 Subtheme: Emotional Suffering

Despite the emotional rewards associated with KC, many parents frequently experience significant emotional suffering. Several papers across both continuous and intermittent KC indicated the responsibility of caring for a fragile infant induces anxiety, fear of neonatal health conditions and fear of touching that hurts their vulnerable infants (Arivabene and Tyrrell 2010; Asmare et al. 2021; Dong et al. 2022; Foong et al. 2023; Jesus et al. 2015; Kourouma et al. 2021; Lewis et al. 2019; Lopes et al. 2020; Maastrup et al. 2018; Mpongwana‐Ncetani et al. 2023; Olsson et al. 2017; Pereira Viana et al. 2018; Suza et al. 2020; Zeng et al. 2023). According to the research conducted by Lopes et al. (2020), fathers in Brazil experience negative feelings of fear and insecurity when attempting KC for the first time. One participant said:

I was afraid at first. I was a little insecure because she was very fragile, and I was afraid of hurting her(Lopes et al. 2020, 4).

This practice can also cause parents to express concerns and emotional distress, leading to feelings of stress due to their unfamiliarity with the neonatal unit environment, medical equipment and constant monitoring that contributes to a sense of uncertainty (Dong et al. 2022; Foong et al. 2023; Mpongwana‐Ncetani et al. 2023; Naloli et al. 2021; Olsson et al. 2017). Furthermore, the research by Foong et al. (2023, 4) in Malaysia revealed that some parents feel embarrassed when exposing their chests to provide KC in an open ward. One mother expressed her concerns, stating that.

I feel exposed. Embarrassed. Yes, screens provide some privacy, but I still don't feel comfortable.

Although KC is often described as emotionally rewarding in experience, fathers have also reported emotional suffering during KC implementation. Five studies reported that fathers commonly expressed feelings of fear and hesitation about touching and fear of potentially hurting their infant because they were concerned about their ability to perform KC correctly (Dong et al. 2022; Jesus et al. 2015; Lopes et al. 2020; Olsson et al. 2017; Zeng et al. 2023). One father's statement,

I did not dare to touch her. It was very small and skinny. I was afraid to pick up and hurt. This for a father is frustrating(Jesus et al. 2015, 8545).

Additionally, studies by Dong et al. (2022) in Sweden and Olsson et al. (2017) in Australia indicated that fathers became anxious and nervous when confronted with unfamiliar equipment, values on the screen of the infant's monitoring and the sound of monitor alarms that made them fear of something going wrong and lead to potential emergencies or complications in their infant's health condition.

##### 5.3.2.2 Subtheme: Physical Burden

The review highlighted the physically demanding nature of KC, which presents a significant challenge for parents. According to various research findings, many parents engaging in continuous or intermittent KC reported feeling fatigued, exhausted and uncomfortable (Blomqvist et al. 2011; Dong et al. 2022; Foong et al. 2023; Noren et al. 2018; Olsson et al. 2017; Pereira Viana et al. 2018). Particularly while continuously KC, they experienced back pain from the prolonged periods of holding their infant in the KC position (Mpongwana‐Ncetani et al. 2023; Naloli et al. 2021). Likewise, a study in South Africa stated that parents who continuously KC also experienced fatigue due to sustained prolonged posture (Ndou et al. 2021, 101). One parent described it as follows:

I feel tired on my vertebral column as if I had been carrying a heavy load on my back, but my focus is on helping my babies to grow.

Moreover, implementing KC led to significant sleep disruption and demanded continuous practice, contributing to sleep deprivation among parents (Mpongwana‐Ncetani et al. 2023; Olsson et al. 2017). These physical burdens can result in parents' reluctance to engage in the practice of KC. Furthermore, KC can also impose a physical burden and drain on fathers, leading to fatigue, exhaustion and backaches when they are required to maintain the KC position for an extended period (Blomqvist et al. 2011; Dong et al. 2022; Olsson et al. 2017). One father described.

We are so terribly tired all the time, and when it got dark, and we went to bed we did not know if it was day or night. All parents we encountered were also completely worn out, so the atmosphere there was quite out of the ordinary(Blomqvist et al. 2011, 1992).

Similarly, two studies conducted in Sweden stated that while fathers were committed to providing KC, the physical strain resulting from uncomfortable positions has been identified as a significant obstacle, impeding their ability to obtain sufficient sleep and rest (Blomqvist et al. 2011; Olsson et al. 2017). The prolonged physical strain associated with KC can influence fathers, leading to sleep pattern disturbances and further increased feelings of exhaustion, which can have a negative impact on their ability to care for their infant effectively.

#### 5.3.3 Theme Three: Roadblocks and Difficulties in Adopting Kangaroo Care

The implementation of intermittent and continuous KC is not without barriers and difficulties. Findings from 19 papers contributed to this theme, elucidated under three subthemes of barriers: healthcare system and professionals' barriers, cultural and social barriers, physical barriers and medical issues that hinder widespread and effective adoption of KC.

##### 5.3.3.1 Subtheme: Healthcare System and Professionals' Barriers

Fourteen studies identified the challenges of inadequate facilities and lack of the necessary infrastructure as significant barriers to embracing both intermittent and continuous KC. Many parents encountered difficulties due to a lack of appropriate facilities and structural environment in the neonatal units, including a lack of private spaces, limited resources at facilities and insufficient food provision that deterred parents from actively participating in KC (Asmare et al. 2021; Blomqvist et al. 2011; Esewe and Phetlhu 2022; Foong et al. 2023; Kourouma et al. 2021; Lopes et al. 2020; Naloli et al. 2021; Ndou et al. 2021; Noren et al. 2018; Olsson et al. 2017; Salimi et al. 2014; Saltzmann et al. 2021; Suza et al. 2020; Yue et al. 2020). The loss of privacy and exposure experienced during KC often leads to feelings of unprotection and discomfort (Yue et al. 2020). One parent from Indonesia expressed,

There are many mothers around the room who have to share places to do KMC, and lack of privacy makes me uncomfortable(Suza et al. 2020, 2278).

Another mother in Uganda practising continuous KC highlighted the shortage of comfortable beds and chairs is affecting the difficulty of sleeping, stating,

Here we have limited space, so mothers tend to squeeze among themselves because the chairs are placed in between the small beds, and since we have no beds, we use the chairs for sleeping on(Naloli et al. 2021, 10).

During the implementation of KC, healthcare providers play a critical role in facilitating and encouraging parents to practice. One frequently mentioned barrier is inadequate human resources, increased workload due to staff shortage and insufficient support from healthcare professionals (Esewe and Phetlhu 2022; Foong et al. 2023; Jesus et al. 2015; Mpongwana‐Ncetani et al. 2023; Naloli et al. 2021; Salimi et al. 2014; Yue et al. 2020). For example, a study conducted in Nigeria, an LMIC, revealed that a lack of human resources and insufficient information for parental engagement in KC is more challenging. The study quoted a parent stating,

The main problem is lack of nurses; government should employ more nurses here are not much(Esewe and Phetlhu 2022, 73).

Similarly, a study in Uganda, an LIC, reported that insufficient education, training and supportive services contributed to the failure to provide KC. As one parent described,

The thing is that the training is lacking, and most of us don't know how to tie the baby because we don't know how many clothes we need and how to place the baby on the chest before trying them because the health workers don't teach us(Naloli et al. 2021, 9).

Three studies emphasise that parents are concerned about the financial struggle associated with the resources needed to provide KC, particularly among those from lower socio‐economic backgrounds who are committed to consistent KC practices. The sustained implementation of KC entails additional costs related to prolonged hospitalisation, transportation, accommodation near the hospital, food expenses and loss of income resulting from time away from work (Esewe and Phetlhu 2022; Lewis et al. 2019; Yue et al. 2020). These financial burdens pose barriers and contribute to negative impacts that increase stress, consequently affecting the ability of parents to maintain KC effectively and consistently.

##### 5.3.3.2 Subtheme: Cultural and Social Barriers

Families' acceptance, cultural beliefs and social norms were also mentioned as hindering the adoption of KC. The findings reported that in some cultural beliefs in Cotte d'Ivoire, Nigeria, South Africa, China and Uganda, where KC conflicted with traditional practice and difficulties in community acceptance led to resistance from family members and challenges in the continuation of its implementation (Doukouré et al. 2022; Esewe and Phetlhu 2022; Foong et al. 2023; Mpongwana‐Ncetani et al. 2023; Naloli et al. 2021; Yue et al. 2020). A study conducted by Kourouma et al. (2021) in Cote d'Ivoire found that specific cultural practices conflicted with the concept of KC due to a lack of community awareness, resistance from fathers and the belief that carrying a baby on the chest was not well‐perceived as culturally acceptable. In one instance, a study in Uganda reported that certain community members perceived KC negatively, stating one mother,

But my tribe as a Mugwere the people out there tend to look at Kangaroo as a bad practice, and it is viewed wrongly from society(Naloli et al. 2021, 12).

Furthermore, Yue et al. (2020) in China and Foong et al. (2023) findings in Malaysia confirmed that strict adherence to traditional Chinese cultural norms, particularly restricting visitation and social interaction during the postpartum confinement period, impedes engagement in KC.

##### 5.3.3.3 Subtheme: Physical Barriers and Medical Issues

The literature has identified several physical challenges and medical concerns as significant barriers contributing to consistent KC implementation. Physical challenges make prolonged KC practice difficult for parents to sustain. Parents may experience fatigue, sweat and postpartum pain complications following caesarean sections, which were considered a hindrance to their ability for extended KC practice (Asmare et al. 2021; Blomqvist et al. 2011; Dong et al. 2022; Doukouré et al. 2022; Lewis et al. 2019; Naloli et al. 2021). A mother in Ethiopia expressed,

I had surgery, and I felt a slight pain when her leg touched the wound(Asmare et al. 2021, 65).

Furthermore, medical concerns regarding the clinical conditions of infants also pose difficulties for KC. Eight studies indicate that parents are concerned regarding the safety and health status of infants (Asmare et al. 2021; Lewis et al. 2019; Mpongwana‐Ncetani et al. 2023; Naloli et al. 2021; Olsson et al. 2017; Salimi et al. 2014; Saltzmann et al. 2021; Yue et al. 2020), inhibiting parents' willingness to participate in KC. The studies detail how fear is related to neonatal medical conditions due to the infant's size, ability to breathe, presence of medical devices (e.g., tracheal, intra‐vascular line), risk of dislodging equipment and wires connected to the infant for monitoring, thereby inducing stress and hesitation when considering participation in KC.

#### 5.3.4 Theme Four: Building Bridges to Encourage and Support Kangaroo Care

Despite the challenges involved, several factors have been identified as facilitating, supporting and the feasibility of intermittent and continuous KC. A comprehensive analysis of 16 papers contributed to three subthemes informing this overarching theme. The findings underscore the importance of the healthcare system support, social and psychological support and strengthening family support. This information significantly influences how facilitators support and motivate parents to overcome barriers encountered in successful KC practice.

##### 5.3.4.1 Subtheme: Healthcare System Support

This review found that key factors, including supportive policies, strong hospital administration leadership and comprehensive staff training, were crucial in promoting and motivating parental adoption of KC (Kourouma et al. 2021; Yue et al. 2020). Fourteen studies underscore the role of healthcare providers in encouraging and facilitating parental involvement in implementing KC. The provision of educational materials, which include instructing parents on caring for their babies, providing knowledge about the benefits and understanding of KC, and offering emotional support from healthcare professionals, is a critical facilitator for parents in successfully performing KC (Asmare et al. 2021; Blomqvist et al. 2011; Foong et al. 2023; Kourouma et al. 2021; Lewis et al. 2019; Lopes et al. 2020; Maastrup et al. 2018; Mpongwana‐Ncetani et al. 2023; Naloli et al. 2021; Ndou et al. 2021; Olsson et al. 2017; Pereira Viana et al. 2018; Saltzmann et al. 2021; Yue et al. 2020). A Swedish study further highlighted that the healthcare providers offered were helpful and enhanced parental confidence and security during KC practice (Olsson et al. 2017). Furthermore, several studies also emphasised that creating a conducive environment within neonatal units, including access to adequate private space, quiet rooms, comfortable beds and chairs, proper laundry facilities and access to television and kitchen amenities for food preparation, was an important factor in promoting parental acceptance and uptake of KC (Asmare et al. 2021; Foong et al. 2023; Noren et al. 2018; Olsson et al. 2017).

##### 5.3.4.2 Subtheme: Social and Psychological Support

Several studies point out that psychological and emotional support from social groups, encompassing peer support groups, counselling services and social media communities, was identified as a critical component in establishing a supportive community of KC (Lewis et al. 2019; Mpongwana‐Ncetani et al. 2023; Naloli et al. 2021; Noren et al. 2018; Olsson et al. 2017; Yue et al. 2020). The involvement of fellow parents who have undergone similar experiences in providing reassurance, practical advice and a sense of shared understanding is essential and instrumental in encouraging and sustaining the practice of KC. A study conducted in South Africa indicates that the role of parental support groups in furnishing information, emotional support and sharing experiences with other parents is to create a nurturing environment, help parents manage their emotional burdens, provide solace and instil a sense of hope. A mother expressed,

I spoke with other mothers who were not having premature babies for the first time, they would encourage me with their other kids where 5 or 7 years old and they were fine now, that gave me hope(Mpongwana‐Ncetani et al. 2023, 8).

Similarly, a study conducted in Uganda (Naloli et al. 2021) explained the importance of peer counselling in reminding and encouraging parents to engage in KC despite the challenges they encounter.

##### 5.3.4.3 Subtheme: Strengthening Family Support

Eight studies have emphasised the significance of family support; the involvement of partners in taking turns practising, grandparents and other relatives taking care of the other children, and assistance with household chores were all important in facilitating emotional and practical support to parents for KC utilisation (Arivabene and Tyrrell 2010; Asmare et al. 2021; Blomqvist et al. 2011; Naloli et al. 2021; Ndou et al. 2021; Noren et al. 2018; Olsson et al. 2017; Yue et al. 2020). Three studies in Sweden (Blomqvist et al. 2011; Noren et al. 2018; Olsson et al. 2017) highlight the importance of family support. The findings found that active partner involvement, with the presence of both parents in the hospital, significantly facilitates the practice. This enabled them to take turns providing continuous KC, thereby allowing others to have a period of rest. As one father said,

We've arranged routines to achieve the right balance. I take the evenings, and she takes the nights and mornings, and like that, we organise it so we both get the same amount of time(Olsson et al. 2017).

Additionally, parents also value the support offered by other relatives (Arivabene and Tyrrell 2010; Blomqvist et al. 2011; Naloli et al. 2021; Ndou et al. 2021; Olsson et al. 2017), such as taking care of other siblings, housekeeping and bringing food, making it possible for the parents to focus and dedicate more time to provide KC. One mother from Uganda stated,

I have enough time, and I can do kangaroo because there is another person who helps me do the kangaroo and also, they help me do other things like washing clothes and cooking food(Naloli et al. 2021, 8).

## 6 Discussion

It has been two decades since the WHO published its recommendations on KMC (WHO 2003).

Since then, there has been a myriad of research exploring its benefits, including decreased mortality and morbidity, increased breastfeeding rates, a cost‐effective method of thermoregulation and a way of protecting parental mental health (Boundy et al. 2016; WHO 2022a; WHO 2022b; Sivanandan and Sankar 2023); but less exploring parental experiences in different health systems and particularly in LICs. As expected, the findings of this review emphasised parental fulfilment from KC, which is universally experienced among diverse healthcare systems, regardless of the different socio‐economic and healthcare contexts of whether the KC was continuous or intermittent and whether the mother or father practised it. This review identified studies describing parental experiences with intermittent KC, a more common practice in HICs. Several studies also investigated parental experiences related to continuous KC, reflecting its widespread adoption in LMICs and LICs. Both intermittent and continuous KC were universally associated with unique positive emotional fulfilment and rewarding experiences among parents. These practices enhance emotional bonding, provide empowering experiences and foster increased confidence in parental caregiving roles. This is consistent with the findings of other research (Mu et al. 2019).

Interestingly, included studies capturing unique parental experiences, particularly among fathers, were conducted in UMICs and HICs (Garnica‐Torres et al. 2024). This may indicate that increasing fathers' involvement, like KC in general, is more prevalent in HICs (Noergaard et al. 2017; Lawal et al. 2023) or simply that research in LICs tends to focus on survival rates (WHO Immediate KMC Study Group 2021). This review does, however, support the importance of fathers in using intermittent KC, establishing their own role as a parent and supporting mothers in carrying it out. There is some suggestion that, in certain LICs, fathers are unwelcome in the maternity unit, with a description of the healthcare workers scaring fathers away (Naloli et al. 2021), which is beyond the scope of this review but needs further investigation.

While both intermittent and continuous KC led to parental fulfilment and hardship, the degree of parental emotional fulfilment varies depending on systemic healthcare support, which in turn depends on well‐motivated and well‐educated neonatal nurses/healthcare providers. There is a well‐documented deficit in neonatal nurses in HICs, which was noted by participants in the reviewed material but which is much worse in MICs and LICs (Bagwell et al. 2024). Generally, HICs possess more advanced healthcare systems and greater financial resources, including equipment and personnel. However, it should not be forgotten that continuous KC was developed in LICs, where healthcare workers and equipment were scarce, to increase the survival of preterm babies (Rey and Martinez 1983). In LICs, where the lack of working incubators often means that the parent is the only means of thermoregulating the neonate, KC is not an intermittent chosen task but can be a full‐time job, seeing mothers isolated from their families and in an environment where basics such as food and even a chair or bed may not be available. The emotional burden was more pronounced in limited systemic healthcare support settings. The physical hardship, which mothers seem glad to endure for their babies, may, however, in these circumstances lead to lower rather than higher breastfeeding rates, one of the well‐documented advantages of KC (Kourouma et al. 2021; Cattaneo et al. 2018; Dhage et al. 2023). Furthermore, cultural norms and social barriers also serve an important role in accepting KC in LMICs. Across specific cultural contexts such as China and Bangladesh, parental adherence to traditional beliefs about infant care may conflict with the principle of KC, subsequently resulting in resistance from family members and grandparents and limited acceptance within the community that influences decision‐making in performing KC (Charpak and Gabriel Ruiz‐Peláez 2006; Hunter et al. 2014; Yue et al. 2020).

In comparison, in high‐resource settings, the barriers to intermittent KC are typically related to rigid hospital protocols that restrict visiting hours, limit access to the neonatal unit or prioritise medical technology. These barriers are often logistical, not driven by basic survival needs, as in low‐resource settings. They are linked to physical challenges and medical condition concerns of the mother and infant that can impede parent desire for KC adoption (Chan et al. 2015; Smith et al. 2017; Suitor 2022).

KC offers substantial emotional rewards; however, one of the key findings from our meta‐synthesis was that hardships involved potentially affect parents' willingness to provide KC. These hardships universally encompass emotional suffering, including anxiety, concern about neonatal health conditions and fear of hurting their vulnerable infant. However, all parents reported that with practice, they gradually developed confidence, which led to a reduction in anxiety and stress levels (Cai et al. 2022). Even in HICs, both mothers and fathers experienced fatigue and exhaustion, muscle strain associated with prolonged posture in KC sessions and sleep deprivation. They reported emotional fatigue from the pressure to engage in extended, uninterrupted sessions of continuous KC, emotional burdens from unfamiliarity with the neonatal unit environment and advanced medical technology (Anderzén‐Carlsson et al. 2014; Cattaneo et al. 2018). Hardship experiences are universal aspects of the intermittent and continuous KC experience, but this is particularly pronounced in low‐resource settings where there were not enough beds and mothers had to sleep in chairs. Parents collectively prioritise their infant's well‐being and health benefits over their distress and discomfort. This finding underscores the crucial necessity of addressing these challenges and physical burdens experienced to increase the successful adoption and sustainability of KC.

More similarities than differences were found in the experiences of mothers and fathers practising intermittent KC. However, there is evidence, mainly from HICs, that fathers find it difficult to find their place in a neonatal unit where their desire to co‐parent is balanced with their need, or perceived need, to provide for the mother, both emotionally and financially (Fisher et al. 2018; Noergaard et al. 2017). KC, as evidenced in this review, is a way of allowing them to co‐parent in a meaningful way. Their participation has the effect of allowing them to remove some of the burden of discomfort from the mothers and also in finding their identity as a father (Dong et al. 2022).

This meta‐synthesis also highlights that it is imperative to recognise the importance of building bridges to strongly encourage and support parents engaging in KC. There are good ergonomic reasons why KC is practised differently in different settings, but whether intermittent or continuous, it has been shown to be effective. In either case, and whatever the setting, the role of the healthcare system is critical in providing support, including the formulation of hospital policies, the development of protocols and the provision of staff training. Ideally, the healthcare system should also prioritise creating a suitable environment and facilities within neonatal units and providing necessary financial resources, but this is difficult to achieve in low‐income settings. Furthermore, healthcare professionals played an important role as informants and facilitators for parents, thereby increasing parental readiness to participate in KC for their infants (Sjömar et al. 2023). In high‐resource settings, some hospitals have integrated KC into standard newborn care protocols and provided supportive environments and dedicated staff. These essential facilitators are crucial for helping parents to effectively practise and sustain the successful implementation of KC (Heinemann et al. 2013; Suitor 2022). In LMICs, continuous KC is frequently adopted as an alternative to incubator care; this means offering adequate support from healthcare providers, sufficient facilities and basic needs such as food, rest and transportation assistance are crucial to enable parents to continue practising KC (Kinshella et al. 2021; Seidman et al. 2015; Sjömar et al. 2023). Family support, involving partners, grandparents and relatives, also facilitates the practice. Notably, social and family support with father participation in taking turns providing KC, encouragement, taking care of other children, and performing household chores all significantly contribute to enabling parents to continue implementing KC (Bayo et al. 2022; Mu et al. 2019; Smith et al. 2017). Furthermore, fostering a positive attitude and experience among parents regarding KC is equally important, as it provides strong social and psychological support. For instance, peer support from other mothers who shared their experiences, empowering parents to provide KC as they learn from one another, and emotional support can be instrumental in promoting societal acceptance and enhancing the adoption of KC (Sjömar et al. 2023). Based on the findings, the consistency and effectiveness of KC across various healthcare systems should be enhanced. The healthcare system is crucial in addressing the barriers and developing tailored multifaceted support strategies that accommodate the specific needs and unique challenges faced by parents in different healthcare settings. Furthermore, healthcare providers, especially nurses, serve a critical role as facilitators of KC and significantly contribute to informing knowledge about the benefits of KC, actively encouraging and supporting parents in its implementation. These strategies can contribute to the widespread adoption and sustainability of KC across diverse healthcare systems.

### 6.1 Strengths and Limitations of This Review

The strength of the meta‐synthesis comes from the expertise of the authors. Two authors have experience in neonatal nursing and one in midwifery. They have worked on three continents and are all experienced academics. Inevitably, any review has some limitations. Although the search covered seven databases, search limiters only included studies published in English, peer‐reviewed articles and within specific publication periods. Grey literature was not sourced in the search, potentially leading to the omission of relevant research. A systematic and rigorous methodological approach to searching and data analysis resulted in a high‐quality meta‐synthesis. Three independent reviewers participated in all steps encompassing study selection, quality appraisal, extraction and synthesis to ensure robustness and reliability. Furthermore, the predominance of studies from UMICs could have affected the broader applicability and generalisability to other countries. Lastly, by definition, only the experiences of those parents who experienced KC were included. This meta‐synthesis was unable to consider any data on those unwilling or unable to participate in KC, whatever their reason.

## 7 Conclusion

This meta‐synthesis has identified the universal value of KC across various healthcare systems, revealing the diverse parental experiences when providing KC. Both mothers and fathers report fulfilment experiences derived from KC, strengthening bonding and empowering parental roles, which underscores the universal emotional reward of the practice. However, parental experiences differ based on healthcare systems, where hardships are more pronounced in low‐resource settings. Despite these difficulties, the parents involved demonstrate remarkable resilience, willingness and strong commitment to participate in KC driven by their belief that KC potentially improves the health outcomes of infants. Their experiences are shaped by various barriers and facilitators depending on the infrastructure, system and socio‐cultural factors in high‐ and low‐resource settings that influenced KC implementation across diverse healthcare systems. Importantly, addressing hardships and barriers parents encounter while building support will facilitate parental involvement. It can be concluded that implementing KC requires healthcare system support, ensuring resource availability, support from healthcare providers and social and family support. This holistic support can foster sustained parental involvement and promote the widespread implementation of KC in neonatal units, ultimately enhancing both parental well‐being and infant health outcomes.

### 7.1 Implications for Practice

This meta‐synthesis presents valuable insight and a comprehensive understanding of parents' experiences with KC. These findings have the potential to impact clinical practice significantly, inform policy and guideline development, improve practice and enhance the effectiveness of KC in the neonatal unit, thus substantially impacting neonatal care. Acknowledging and overcoming the barriers and leveraging the facilitators, the healthcare system and professionals play crucial roles in providing more empathetic and tailored support to empower parents. This includes providing information about KC, education, access to resources and basic amenities, and offering emotional support to enhance efficacy and facilitate parental participation in KC. Additionally, future research and further identification of evidence‐based practice are necessary for widespread adoption, ultimately improving KC utilisation and neonatal health outcomes.

## Author Contributions

All authors made substantial contributions to conception and design, or acquisition of data, or analysis and interpretation of data; involved in drafting the manuscript or revising it critically for important intellectual content; given final approval of the version to be published. Each author should have participated sufficiently in the work to take public responsibility for appropriate portions of the content; agreed to be accountable for all aspects of the work in ensuring that questions related to the accuracy or integrity of any part of the work are appropriately investigated and resolved. All authors have contributed to the conception and writing of the paper. The review protocol was written by S.P. and received approval from the supervisors M.H. and B.B. S.P. was responsible for developing the search strategies, conducting all database searches, initially screening the literature, performing data analysis and interpretation and drafting the manuscript. The second author (M.H.) reviewed the full text of the included papers and screened them for meeting inclusion or exclusion criteria. A third reviewer (B.B.) was available to provide an independent opinion of any dispute decisions. All authors have independently screened and collaboratively involved in the analysis and interpretation of data, critically appraising the content, revising the manuscript and approving the final version for publication.

## Ethics Statement

This study was approved by the Faculty of Medicine, Health and Life Sciences Research Ethics Committee (Faculty REC) at Queen's University Belfast (Approval No. MHLS 24_87).

## Conflicts of Interest

The authors declare no conflicts of interest.

## Supporting information




Appendix S1.

## Data Availability

The data that supports the findings of this study are available in the Supporting Information of this article.

## References

[jan17058-bib-0001] Anderzén‐Carlsson, A. , Z. C. Lamy , and M. Eriksson . 2014. “Parental Experiences of Providing Skin‐To‐Skin Care to Their Newborn Infant—Part 1: A Qualitative Systematic Review.” International Journal of Qualitative Studies on Health and Well‐Being 9, no. 1: 24906. 10.3402/qhw.v9.24906. 25319746 PMC4197399

[jan17058-bib-0002] Arivabene, J. C. , and M. A. R. Tyrrell . 2010. “Kangaroo Mother Method: Mothers' Experiences and Contributions to Nursing.” Revista Latino‐Americana de Enfermagem 18, no. 2: 262–268. 10.1590/s0104-11692010000200018. 20549127

[jan17058-bib-0003] Asmare, M. G. , R. Murugan , and M. Adimasu . 2021. “Perceived Enablers and Barriers of Kangaroo Mother Care Among Mothers and Nurses in Tikur Anbessa Specialized Hospital, Addis Ababa, Ethiopia: A Qualitative Study.” Iranian Journal of Neonatology 12, no. 4: 63–69. 10.22038/ijn.2021.53431.1976.

[jan17058-bib-0004] Bagwell, G. A. , S. K. Cesario , D. Fraser , C. Kenner , and K. Walker . 2024. “Breaking the Cycle of Nursing Chaos: The Need to Address the Nursing Shortage.” Advances in Neonatal Care 23, no. 6: 495–498. 10.1097/ANC.0000000000001126 38038669

[jan17058-bib-0005] Bayo, P. , G. Alobo , C. Sauvé , and G. T. Feyissa . 2022. “Mothers' Perceptions of the Practice of Kangaroo Mother Care for Preterm Neonates in Sub‐Saharan Africa.” JBI Evidence Synthesis 20, no. 2: 297–347. 10.11124/jbies-20-00435. 34171891

[jan17058-bib-0006] Blomqvist, Y. T. , C. Rubertsson , E. Kylberg , K. Joreskog , and K. H. Nyqvist . 2011. “Kangaroo Mother Care Helps Fathers of Preterm Infants Gain Confidence in the Paternal Role.” Journal of Advanced Nursing 68, no. 9: 1988–1996. 10.1111/j.1365-2648.2011.05886.x. 22111919

[jan17058-bib-0007] Boundy, E. O. , R. Dastjerdi , D. Spiegelman , et al. 2016. “Kangaroo Mother Care and Neonatal Outcomes: A Meta‐Analysis.” Pediatrics 137, no. 1: e20152238. 10.1542/peds.2015-2238. 26702029 PMC4702019

[jan17058-bib-0008] Braun, V. , and V. Clarke . 2019. “Reflecting on Reflexive Thematic Analysis.” Qualitative Research in Sport, Exercise and Health 11, no. 4: 589–597. 10.1080/2159676X.2019.1628806.

[jan17058-bib-0009] Cai, Q. , D. Q. Chen , H. Wang , et al. 2022. “What Influences the Implementation of Kangaroo Mother Care? An Umbrella Review.” BMC Pregnancy and Childbirth 22, no. 1: 851. 10.1186/s12884-022-05163-3. 36401193 PMC9675107

[jan17058-bib-0010] Cattaneo, A. , A. Amani , N. Charpak , et al. 2018. “Report on an International Workshop on Kangaroo Mother Care: Lessons Learned and a Vision for the Future.” BMC Pregnancy and Childbirth 18, no. 1: 1–10. 10.1186/s12884-018-1819-9. 29769056 PMC5956892

[jan17058-bib-0011] Chan, G. J. , A. S. Labar , S. Wall , and R. Atun . 2015. “Kangaroo Mother Care: A Systematic Review of Barriers and Enablers.” Bulletin of the World Health Organization 94, no. 2: 130–141. 10.2471/blt.15.157818. 26908962 PMC4750435

[jan17058-bib-0012] Chang, A. Y. , K. Cowling , A. E. Micah , et al. 2019. “Past, Present, and Future of Global Health Financing: A Review of Development Assistance, Government, Out‐Of‐Pocket, and Other Private Spending on Health for 195 Countries, 1995–2050.” Lancet 393, no. 10187: 2233–2260. 10.1016/S0140-6736(19)30841-4. 31030984 PMC6548764

[jan17058-bib-0013] Charpak, N. , and J. Gabriel Ruiz‐Peláez . 2006. “Resistance to Implementing Kangaroo Mother Care in Developing Countries, and Proposed Solutions.” Acta Paediatrica 95, no. 5: 529–534. 10.1111/j.1651-2227.2006.tb02279.x. 16825131

[jan17058-bib-0014] Conde‐Agudelo, A. , and J. L. Díaz‐Rossello . 2016. “Kangaroo Mother Care to Reduce Morbidity and Mortality in Low Birthweight Infants.” Cochrane Database of Systematic Reviews 2016, no. 8: CD002771. 10.1002/14651858.cd002771.pub4. 27552521 PMC6464509

[jan17058-bib-0015] Critical Appraisal Skills Programme (CASP) . 2018. “CASP Appraisal Qualitative Checklist.” https://casp‐uk.net/checklists/casp‐qualitative‐studies‐checklist.pdf.

[jan17058-bib-0016] Dhage, V. D. , A. Rannaware , and S. G. Choudhari . 2023. “Kangaroo Mother Care for Low‐Birth‐Weight Babies in Low and Middle‐Income Countries: A Narrative Review.” Cureus 15, no. 4: e38355. 10.7759/cureus.38355. 37274008 PMC10232296

[jan17058-bib-0017] Dong, Q. , M. Steen , D. Wepa , and A. Eden . 2022. “Exploratory Study of Fathers Providing Kangaroo Care in a Neonatal Intensive Care Unit.” Journal of Clinical Nursing. 10.1111/jocn.16405. PMC12581099 35712782

[jan17058-bib-0018] Doukouré, D. , K. R. Kourouma , M. L. A. Yacé , et al. 2022. “Acceptability of the Kangaroo Mother Care at the University Hospital of Treichville in Côte D'ivoire.” Journal of Public Health in Africa 13, no. 3: 2165. 10.4081/jphia.2022.2165. 36337678 PMC9627761

[jan17058-bib-0019] Esewe, R. E. , and R. D. Phetlhu . 2022. “Challenges of Uptake of Kangaroo Mother Care by Parents of Preterm and Low Birth Weight Infants in Edo State, Nigeria.” African Journal of Reproductive Health 26, no. 2: 68–79. 10.29063/ajrh2022/v26i2.7. 37584998

[jan17058-bib-0020] Finfgeld‐Connett, D. 2010. “Generalizability and Transferability of Meta‐Synthesis Research Findings.” Journal of Advanced Nursing 66, no. 2: 246–254. 10.1111/j.1365-2648.2009.05250.x. 20423407

[jan17058-bib-0021] Fisher, D. , M. Khashu , E. A. Adama , et al. 2018. “Fathers in Neonatal Units: Improving Infant Health by Supporting the Baby‐Father Bond and Mother‐Father Coparenting.” Journal of Neonatal Nursing 24, no. 6: 306–312.

[jan17058-bib-0022] Foong, W. C. , S. C. Foong , J. J. Ho , et al. 2023. “Exploring Factors Influencing the Uptake of Kangaroo Mother Care: Key Informant Interviews With Parents.” BMC Pregnancy and Childbirth 23, no. 1: 706. 10.1186/s12884-023-06021-6. 37789260 PMC10548712

[jan17058-bib-0023] Garnica‐Torres, Z. , G. B. Dias , and P. J. da Silva . 2024. “A Systematic Review of Fatherhood and Kangaroo Care in the NICU.” Children and Youth Services Review 157: 107417. 10.1016/j.childyouth.2023.107417.

[jan17058-bib-0024] Gunay, U. , and D. Coskun Simsek . 2021. “Emotions and Experience of Fathers Applying Kangaroo Care in the Eastern Anatolia Region of Turkey: A Qualitative Study.” Clinical Nursing Research 30, no. 6: 840–846. 10.1177/1054773820937479. 32613856

[jan17058-bib-0025] Heinemann, A. B. , L. Hellström‐Westas , and K. Hedberg Nyqvist . 2013. “Factors Affecting Parents' Presence With Their Extremely Preterm Infants in a Neonatal Intensive Care Room.” Acta Paediatrica 102, no. 7: 695–702. 10.1111/apa.12267. 23590800

[jan17058-bib-0026] Hunter, E. C. , J. A. Callaghan‐Koru , A. Al Mahmud , et al. 2014. “Newborn Care Practices in Rural Bangladesh: Implications for the Adaptation of Kangaroo Mother Care for Community‐Based Interventions.” Social Science & Medicine 122: 21–30. 10.1016/j.socscimed.2014.10.006. 25441314

[jan17058-bib-0027] Jesus, N. C. , B. D. Vieira , V. H. Alves , D. P. Rodrigues , R. D. Souza , and E. D. Paiva . 2015. “The Experience of the Kangaroo Method: The Perception of the Father.” Journal of Nursing Ufpe Online 9, no. 7: 8542–8550. 10.5205/7310.

[jan17058-bib-0028] Kinshella, M. L. W. , T. Hiwa , K. Pickerill , et al. 2021. “Barriers and Facilitators of Facility‐Based Kangaroo Mother Care in Sub‐Saharan Africa: A Systematic Review.” BMC Pregnancy and Childbirth 21, no. 1: 176. 10.1186/s12884-021-03646-3. 33663415 PMC7934357

[jan17058-bib-0029] Kourouma, K. R. , M. L. Agbré‐Yacé , D. Doukouré , et al. 2021. “Barriers and Facilitators to Kangaroo Mother Care Implementation in Cote d'Ivoire: A Qualitative Study.” BMC Health Services Research 21, no. 1: 1211. 10.1186/s12913-021-07086-9. 34753464 PMC8576306

[jan17058-bib-0030] Lawal, T. V. , D. I. Lawal , and O. J. Adeleye . 2023. “Determinants of Kangaroo Mother Care Among Low‐Birth‐Weight Infants in Low Resource Settings.” PLOS Global Public Health 3, no. 9: e0002015. 10.1371/journal.pgph.0002015. 37699007 PMC10497168

[jan17058-bib-0031] Lewis, T. P. , K. G. Andrews , E. Shenberger , et al. 2019. “Caregiving Can Be Costly: A Qualitative Study of Barriers and Facilitators to Conducting Kangaroo Mother Care in a US Tertiary Hospital Neonatal Intensive Care Unit.” BMC Pregnancy and Childbirth 19, no. 1: 227. 10.1186/s12884-019-2363-y. 31272398 PMC6610951

[jan17058-bib-0032] Li, L. , F. Ji , Y. Wang , et al. 2023. “The Clinical Experience of Early Skin‐To‐Skin Contact Combined With Non‐Nutritive Comfort Sucking in Mothers of Preterm Infants: A Qualitative Study.” BMC Pregnancy and Childbirth 23, no. 1: 281. 10.1186/s12884-023-05581-x. 37095429 PMC10123578

[jan17058-bib-0033] Lopes, L. L. , A. Vaccari , F. A. Rodrigues , and S. Herber . 2020. “Fathers' Experiences Using the Kangaroo Position With Low‐Birth‐Weight Infants.” Revista de Enfermagem Referência 5, no. 3: 1–6. 10.12707/RV20033.

[jan17058-bib-0034] Maastrup, R. , J. Weis , A. B. Engsig , K. L. Johannsen , and V. Zoffmann . 2018. “‘Now She has Become My Daughter’: Parents' Early Experiences of Skin‐To‐Skin Contact With Extremely Preterm Infants.” Scandinavian Journal of Caring Sciences 32, no. 2: 545–553. 10.1111/scs.12478. 28851054

[jan17058-bib-0036] Mpongwana‐Ncetani, S. , R. Roomaney , and A. Lachman . 2023. “Experiences of Xhosa Women Providing Kangaroo Mother Care in a Tertiary Hospital in the Western Cape, South Africa.” South Africa Journal of Psychology 53, no. 4: 497–508. 10.1177/00812463231193167.

[jan17058-bib-0037] Mu, P. F. , M. Y. Lee , Y. C. Chen , H. C. Yang , and S. H. Yang . 2019. “Experiences of Parents Providing Kangaroo Care to a Premature Infant: A Qualitative Systematic Review.” Nursing and Health Sciences 22, no. 2: 149–161. 10.1111/nhs.12631. 31430017

[jan17058-bib-0038] Muka, T. , M. Glisic , J. Milic , et al. 2019. “A 24‐Step Guide on How to Design, Conduct, and Successfully Publish a Systematic Review and Meta‐Analysis in Medical Research.” European Journal of Epidemiology 35, no. 1: 49–60. 10.1007/s10654-019-00576-5. 31720912

[jan17058-bib-0039] Naloli, M. , L. V. N. Ssenyonga , E. K. Kagoya , J. Nteziyaremye , and R. Nekaka . 2021. “Kangaroo Mother Care: A Qualitative Study on the Practice and Experiences of Mothers of Preterm Neonates in a Tertiary Teaching Hospital in Eastern Uganda.” International Journal for Research in Health Sciences and Nursing 7, no. 11: 1890. 10.1016/j.gendis.2018.01.004. 36817802 PMC9938524

[jan17058-bib-0040] Ndou, N. D. , T. M. Mulaudzi , R. A. Anokwuru , and A. H. Mavhandu‐Mudzusi . 2021. “Kangaroo Mother Care: Lived Experiences of Mothers in Three Hospitals of Limpopo Province, South Africa.” South African Journal of Child Health 15, no. 2: 99–102. 10.7196/sajch.2021.v15i2.01766.

[jan17058-bib-0041] Noergaard, B. , J. Ammentorp , J. Fenger‐Gron , P. E. Kofoed , H. Johannessen , and S. Thibeau . 2017. “Fathers' Needs and Masculinity Dilemmas in a Neonatal Intensive Care Unit in Denmark.” Advances in Neonatal Care 17, no. 4: E13–E22. 28749826 10.1097/ANC.0000000000000395 PMC5533583

[jan17058-bib-0042] Noren, J. , K. H. Nyqvist , C. Rubertsson , and Y. T. Blomqvist . 2018. “Becoming a Mother – Mothers' Experience of Kangaroo Mother Care.” Sexual & Reproductive Healthcare 16: 181–185. 10.1016/j.srhc.2018.04.005. 29804764

[jan17058-bib-0043] Olsson, E. , M. Eriksson , and A. Anderzen‐Carlsson . 2017. “Skin‐To‐Skin Contact Facilitates More Equal Parenthood ‐ A Qualitative Study From Fathers' Perspective.” Journal of Pediatric Nursing 34: e2–e9. 10.1016/j.pedn.2017.03.004. 28364962

[jan17058-bib-0045] Pathak, B. G. , B. Sinha , N. Sharma , S. Mazumder , and N. Bhandari . 2023. “Effects of Kangaroo Mother Care on Maternal and Paternal Health: Systematic Review and Meta‐Analysis.” Bulletin of the World Health Organization 101, no. 6: 391. 37265678 10.2471/BLT.22.288977 PMC10225947

[jan17058-bib-0046] Pereira Viana, M. R. , L. A. de Nunes Araujo , M. C. Vitorino Sales , and J. M. Magalhaes . 2018. “Experiences of Premature Mothers Regarding the Kangaroo Mother Method.” Revista de Pesquisa: Cuidado é Fundamental Online 10, no. 3: 690–695. 10.9789/2175-5361.2018.v10i3.690-695.

[jan17058-bib-0047] Pradhan, P. , L. Costa , D. Rybski , W. Lucht , and J. P. Kropp . 2017. “A Systematic Study of Sustainable Development Goal (SDG) Interactions.” Earth's Future 5, no. 11: 1169–1179.

[jan17058-bib-0048] Rey, E. S. , and H. G. Martinez . 1983. Manejo Racional del Niño Prematuro [in Spanish]. Curso de Medicina Fetal, Universidad Nacional.

[jan17058-bib-0049] Salimi, T. , M. Khodayarian , M. Bokaie , M. Antikchi , and S. Javadi . 2014. “Mothers' Experiences With Premature Neonates About Kangaroo Care: Qualitative Approaches.” Journal of Pediatric Perspectives 2, no. 1: 75–82. 10.22038/ijp.2014.2124.

[jan17058-bib-0050] Saltzmann, A. M. , K. Sigurdson , and M. Scala . 2021. “Barriers to Kangaroo Care in the NICU: A Qualitative Study Analyzing Parent Survey Responses.” Advances in Neonatal Care 22, no. 3: 261–269. 10.1097/anc.0000000000000907. 34054009

[jan17058-bib-0051] Sandelowski, M. , and J. Barroso . 2007. Handbook for Synthesizing Qualitative Research. Springer.

[jan17058-bib-0052] Seidman, G. , S. Unnikrishnan , E. Kenny , et al. 2015. “Barriers and Enablers of Kangaroo Mother Care Practice: A Systematic Review.” PLoS One 10, no. 5: e0125643. 10.1371/journal.pone.0125643. 25993306 PMC4439040

[jan17058-bib-0053] Sivanandan, S. , and M. J. Sankar . 2023. “Kangaroo Mother Care for Preterm or Low Birth Weight Infants: A Systematic Review and Meta‐Analysis.” BMJ Global Health 8, no. 6: e010728. 10.1136/bmjgh-2022-010728 PMC10254798 37277198

[jan17058-bib-0054] Sjömar, J. , H. Ottesen , G. Banik , et al. 2023. “Exploring Caregivers' Experiences of Kangaroo Mother Care in Bangladesh: A Descriptive Qualitative Study.” PLoS One 18, no. 1: e0280254. 10.1371/journal.pone.0280254. 36689433 PMC9870098

[jan17058-bib-0055] Smith, E. R. , I. Bergelson , S. Constantian , B. Valsangkar , and G. J. Chan . 2017. “Barriers and Enablers of Health System Adoption of Kangaroo Mother Care: A Systematic Review of Caregiver Perspectives.” BMC Pediatrics 17, no. 1: 1–16. 10.1186/s12887-016-0769-5. 28122592 PMC5267363

[jan17058-bib-0056] Stern, C. , Z. Jordan , and A. McArthur . 2014. “Developing the Review Question and Inclusion Criteria.” American Journal of Nursing 114, no. 4: 53–56. 10.1097/01.naj.0000445689.67800.86. 24681476

[jan17058-bib-0057] Suitor, C. 2022. “Kangaroo Mother Care: A Literature Review of Barriers and Facilitators to Implementation in the Neonatal Intensive Care Unit.” Journal of Neonatal Nursing 29, no. 2: 245–252. 10.1016/j.jnn.2022.07.003.

[jan17058-bib-0058] Suza, D. E. , S. Setiawan , and A. Fathi . 2020. “Experience of Indonesian Mothers in Implementing Kangaroo Mother Care During Hospitalization With Low‐Birth‐Weight Neonates.” Indian Journal of Public Health Research & Development 11, no. 3: 2276–2280. 10.37506/ijphrd.v11i3.2725.

[jan17058-bib-0059] Tong, A. , K. Flemming , E. McInnes , S. Oliver , and J. Craig . 2012. “Enhancing Transparency in Reporting the Synthesis of Qualitative Research: ENTREQ.” BMC Medical Research Methodology 12, no. 1: 181. 10.1186/1471-2288-12-181. 23185978 PMC3552766

[jan17058-bib-0060] Walsh, D. , and S. Downe . 2005. “Meta‐Synthesis Method for Qualitative Research: A Literature Review.” Journal of Advanced Nursing 50, no. 2: 204–211. 10.1111/j.1365-2648.2005.03380.x. 15788085

[jan17058-bib-0061] WHO (World Health Organization) . 2003. Reproductive Health. Kangaroo Mother Care: A Practical Guide (No. 1). World Health Organization.

[jan17058-bib-0062] WHO (World Health Organization) . 2015. “WHO Recommendations on Interventions to Improve Preterm Birth Outcomes.” Accessed October 18, 2023. https://iris.who.int/bitstream/handle/10665/183037/9789241508988_eng.pdf?sequence=1. 26447264

[jan17058-bib-0063] WHO (World Health Organization) . 2022a. “WHO Advises Immediate Skin to Skin Care for Survival of Small and Preterm Babies.” Accessed December 6, 2023. https://www.who.int/news/item/15‐11‐2022‐who‐advises‐immediate‐skin‐to‐skin‐care‐for‐survival‐of‐small‐and‐preterm‐babies.

[jan17058-bib-0064] WHO (World Health Organization) . 2022b. “WHO Recommendations for Care of the Preterm or Low‐Birth‐Weight Infant.” https://iris.who.int/bitstream/handle/10665/363697/9789240058262‐eng.pdf?sequence=1. 36449655

[jan17058-bib-0065] WHO (World Health Organization) . 2023. Kangaroo Mother Care: Implementation Strategy for Scale‐Up Adaptable to Different Country Contexts. World Health Organization. https://iris.who.int/handle/10665/367625.

[jan17058-bib-0066] WHO Immediate KMC Study Group . 2021. “Immediate “Kangaroo Mother Care” and Survival of Infants With Low Birth Weight.” New England Journal of Medicine 384, no. 21: 2028–2038. 34038632 10.1056/NEJMoa2026486 PMC8108485

[jan17058-bib-0067] Yue, J. , J. Liu , S. Williams , et al. 2020. “Barriers and Facilitators of Kangaroo Mother Care Adoption in Five Chinese Hospitals: A Qualitative Study.” BMC Public Health 20: 1–11. 10.1186/s12889-020-09337-6. 32791972 PMC7427278

[jan17058-bib-0068] Zeng, X. , L. L. Li , X. Wu , Y. H. Tian , D. D. Gao , and X. J. Hu . 2023. “Qualitative Study on the Experience of Fathers Involved in Kangaroo Care of Premature Infants.” Journal of Neonatal Nursing 29, no. 4: 657–661. 10.1016/j.jnn.2022.12.001.

