# Supplementary material for: Diverse Parental Experiences of Kangaroo Care in Neonatal Units Across Healthcare Systems: A Meta‐Synthesis
Source: J Adv Nurs. 2025 May 19;82(3):1901–27. doi: 10.1111/jan.17058 (PMC12907618; doi:10.1111/jan.17058)
Supplement: Supplementary file 1 — Appendix S1. [file JAN-82-1901-s001.docx]

**Enhancing transparency in reporting the synthesis of qualitative research (ENTREQ)’ statement (Tong et al., 2012)**

(Tong, A., Flemming, K., McInnes, E., Oliver, S., & Craig, J. (2012). Enhancing transparency in reporting the synthesis of qualitative research: ENTREQ. BMC Medical Research Methodology, 12. doi:10.1186/1471-2288-12-181)

**Title: Diverse Parental Experiences of Kangaroo Care in Neonatal Units Across Healthcare Systems: A Systematic Review and Qualitative Meta-Synthesis.**

| **No** | **Item** | **Guide and description** | **Reported on page number (s)** |
| --- | --- | --- | --- |
| 1 | Aim | *State the research question the synthesis addresses.*  The aim of this meta-synthesis was to systematically identify, evaluate, and synthesise the recent evidence on parents’ experiences of Kangaroo Care with their infants in the neonatal unit across health system. | Page 3 |
| 2 | Synthesis methodology | *Identify the synthesis methodology or theoretical framework which underpins the* *synthesis, and describe the rationale for choice of methodology (e.g. meta-ethnography, thematic synthesis, critical interpretive synthesis, grounded theory synthesis, realist synthesis, meta-aggregation, meta-study, framework synthesis).* | Page 5-6 |
| 3 | Approach to searching | *Indicate whether the search was pre-planned (Comprehensive search strategies to seek all available studies) or iterative (to seek all available concepts until theoretical saturation is achieved).*  Search was planned using the PEO framework (Population, Exposure, Outcome), to identify the key search terms and to inform the search strategy. | Page 4 |
| 4 | Inclusion criteria | *Specify the inclusion/exclusion criteria (e.g. in terms of population, language, year limits, type of publication, study type).*  Inclusion criteria   - Studies focusing on women or parents who have experienced Kangaroo Care in the neonatal unit - Qualitative studies or mixed methods studies with reporting of qualitative data - Primary research studies - Full-text articles published in a peer-reviewed journal. - Studies published in English - The publication timeframe was limited between January 2010 and 2024.   Exclusion criteria   - Studies focused on maternity care providers or healthcare professionals' experiences with Kangaroo Care - Studies involved parents providing Kangaroo Care with their infant out of the neonatal unit (operating room and home) - Quantitative methodologies - Secondary studies and non-peer-reviewed literature (e.g., commentary, dissertation, editorials, conference p and review articles) - Secondary studies and non-peer-reviewed literature (e.g., commentary, dissertation, editorials, conference p and review articles) | Page 5 and  Table 2 |
| 5 | Data Sources | *Describe the information sources used (e.g. electronic databases (MEDLINE, EMBASE, CINAHL, psycINFO, Econlit), grey literature databases (digital thesis, policy reports), relevant organisational websites, experts, information specialists, generic web searches (Google Scholar) hand searching, reference lists) and when the searches conducted; provide the rationale for using the data sources.*  A systematic search of five electronic databases was undertaken including: CINAHL, MEDLINE ALL, EMBASE, APA PsycINFO, Maternity & Infant Care, Scopus, and Cochrane Library. | Page 4 |
| 6 | Electronic search strategy | *Describe the literature search (e.g. provide electronic search strategies with population terms, clinical or health topic terms, experiential or social phenomena related terms, filters for qualitative research, and search limits).*  A comprehensive search strategy was formulated using MeSH headings and keywords aligned with the PEO framework, and truncations were tailored appropriately for each database. | Page 4 |
| 7 | Study screening methods | *Describe the process of study screening and sifting (e.g. title, abstract and full text review, number of independent reviewers who screened studies).*  The findings from the search of the electronic databases were directly imported into Covidence systematic review software using the Endnote X9 reference management system, with all duplicate entries removed. The two reviewers selected studies independently by screening titles and abstracts for potential eligibility papers. After that, the full text was carefully considered in detail based on inclusion and exclusion criteria. Any disagreements or conflicts during this process were resolved through discussion with the third reviewer. | Page 6 |
| 8 | Study characteristics | *Present the characteristics of the included studies (e.g. year of publication, country, population, number of participants, data collection, methodology, analysis, research questions).*  The characteristics of the individual studies is documented in the study characteristics table 4 | Page 8-9 and  Table 4 |
| 9 | Study selection results | *Identify the number of studies screened and provide reasons for study exclusion (e.g., for comprehensive searching, provide numbers of studies screened and reasons for exclusion indicated in a figure/flowchart; for iterative searching describe reasons for study exclusion and inclusion based on modifications the research question and/or contribution to theory development).*  The systematic search result is illustrated in the PRISMA flow diagram. | Page 7 and  Figure 1 |
| 10 | Rationale for appraisal | *Describe the rationale and approach used to appraise the included studies or selected findings (e.g. assessment of conduct (validity and robustness), assessment of reporting (transparency), assessment of content and utility of the findings).*  Studies were not excluded based on their CASP score, as they still have a contribution to the review findings. | Page 6 |
| 11 | Appraisal items | *State the tools, frameworks and criteria used to appraise the studies or selected findings (e.g. Existing tools: CASP, QARI, COREQ, Mays and Pope [25]; reviewer developed tools; describe the domains assessed: research team, study design, data analysis and interpretations, reporting).*  CASP tool was used to appraise the included studies. | Page 6 |
| 12 | Appraisal process | *Indicate whether the appraisal was conducted independently by more than one reviewer and if consensus was required.*  Quality appraisal was performed independently by the first and second authors and consensus reached. | Page 6 |
| 13 | Appraisal results | *Present results of the quality assessment and indicate which articles, if any, were weighted/excluded based on the assessment and give the rationale.*  No studies were excluded based on their CASP score. | Table 3 |
| 14 | Data extraction | *Indicate which sections of the primary studies were analysed and how were the data extracted from the primary studies? (e.g. all text under the headings “results /conclusions” were extracted electronically and entered into a computer software).*  Participant quotations and author’s interpretations were analysed. Data was extracted by the first author using NVivo and verified by the second author. | Page 6 |
| 15 | Software | *State the computer software used, if any.*  NVivo | Page 7 |
| 16 | Number of reviewers | *Identify who was involved in coding and analysis.*  The analysis of themes and subthemes was reviewed through discussion and revision among the reviewer team. | Page 6 |
| 17 | Coding | *Describe the process for coding of data (e.g. line by line coding to search for concepts).*  The code development process involved deep and prolonged data immersion, discussion, and comparison of interpretations to capture the meaning of the data and context. Codes were organised into related areas to construct themes through an iterative process. The similarities and differences between the codes were initially organised in a hierarchical structure. | Page 7 |
| 18 | Study comparison | *Describe how were comparisons made within and across studies (e.g. subsequent studies were coded into pre-existing concepts, and new concepts were created when deemed necessary).*  All relevant text from the findings was extracted and entered into the QSR NVivo software. The code development process involved deep and prolonged data immersion, discussion, and comparison of interpretations to capture the meaning of the data and context. | Page 7 |
| 19 | Derivation of themes | *Explain whether the process of deriving the themes or constructs was inductive or deductive.*  The potential themes that emerged were compared within and across studies to answer the review questions. Finally, the final themes were derived inductively and were then summarised to go beyond the findings of the original study. | Page 7 and  Table 5 |
| 20 | Quotations | *Provide quotations from the primary studies to illustrate themes/**constructs, and identify whether the quotations were participant quotations of the author’s interpretation.*  Quotations from participants and authors from primary studies are available in the results. | Page 9-20 |
| 21 | Synthesis output | *Present rich, compelling and useful results that go beyond a summary of the primary studies (e.g. new interpretation, models of evidence, conceptual models, analytical framework, development of a new theory or construct).* | Page 23-24 |
